# Sequence- and structure-selective mRNA m^5^C methylation by NSUN6 in animals

**DOI:** 10.1093/nsr/nwaa273

**Published:** 2020-10-31

**Authors:** Jianheng Liu, Tao Huang, Yusen Zhang, Tianxuan Zhao, Xueni Zhao, Wanying Chen, Rui Zhang

**Affiliations:** MOE Key Laboratory, of Gene Function and Regulation, State Key Laboratory of Biocontrol, School of Life Sciences, RNA Biomedical Institute, Sun Yat-Sen Memorial Hospital, Sun Yat-Sen University, Guangzhou 510275, China; MOE Key Laboratory, of Gene Function and Regulation, State Key Laboratory of Biocontrol, School of Life Sciences, RNA Biomedical Institute, Sun Yat-Sen Memorial Hospital, Sun Yat-Sen University, Guangzhou 510275, China; MOE Key Laboratory, of Gene Function and Regulation, State Key Laboratory of Biocontrol, School of Life Sciences, RNA Biomedical Institute, Sun Yat-Sen Memorial Hospital, Sun Yat-Sen University, Guangzhou 510275, China; MOE Key Laboratory, of Gene Function and Regulation, State Key Laboratory of Biocontrol, School of Life Sciences, RNA Biomedical Institute, Sun Yat-Sen Memorial Hospital, Sun Yat-Sen University, Guangzhou 510275, China; MOE Key Laboratory, of Gene Function and Regulation, State Key Laboratory of Biocontrol, School of Life Sciences, RNA Biomedical Institute, Sun Yat-Sen Memorial Hospital, Sun Yat-Sen University, Guangzhou 510275, China; MOE Key Laboratory, of Gene Function and Regulation, State Key Laboratory of Biocontrol, School of Life Sciences, RNA Biomedical Institute, Sun Yat-Sen Memorial Hospital, Sun Yat-Sen University, Guangzhou 510275, China; MOE Key Laboratory, of Gene Function and Regulation, State Key Laboratory of Biocontrol, School of Life Sciences, RNA Biomedical Institute, Sun Yat-Sen Memorial Hospital, Sun Yat-Sen University, Guangzhou 510275, China

**Keywords:** mRNA m^5^C, NSUN6, RNA modification, protein-RNA complex structure

## Abstract

mRNA m^5^C, which has recently been implicated in the regulation of mRNA mobility, metabolism and translation, plays important regulatory roles in various biological events. Two types of m^5^C sites are found in mRNAs. Type I m^5^C sites, which contain a downstream G-rich triplet motif and are computationally predicted to be located at the 5^′^ end of putative hairpin structures, are methylated by NSUN2. Type II m^5^C sites contain a downstream UCCA motif and are computationally predicted to be located in the loops of putative hairpin structures. However, their biogenesis remains unknown. Here we identified NSUN6, a methyltransferase that is known to methylate C72 of tRNA^Thr^ and tRNA^Cys^, as an mRNA methyltransferase that targets Type II m^5^C sites. Combining the RNA secondary structure prediction, miCLIP, and results from a high-throughput mutagenesis analysis, we determined the RNA sequence and structural features governing the specificity of NSUN6-mediated mRNA methylation. Integrating these features into an NSUN6-RNA structural model, we identified an NSUN6 variant that largely loses tRNA methylation but retains mRNA methylation ability. Finally, we revealed a weak negative correlation between m^5^C methylation and translation efficiency. Our findings uncover that mRNA m^5^C is tightly controlled by an elaborate two-enzyme system, and the protein-RNA structure analysis strategy established may be applied to other RNA modification writers to distinguish the functions of different RNA substrates of a writer protein.

## INTRODUCTION

mRNAs contain numerous modified nucleotides, which have been suggested to play important roles in regulating the fate of mRNA. To date, how writer proteins recognize specific sequence context for mRNA modification is largely unknown. Recently, it has been found that most writer proteins have multiple types of RNA substrates. For example, METTL16 is responsible for both snRNA and mRNA m^6^A modification [1], TRMT6/TRMT61A is responsible for both tRNA and mRNA m^1^A modification [2], and individual PUS proteins are responsible for tRNA and mRNA pseudouridylation [3]. Moreover, for all of these writer proteins, their mRNA substrates have similar features to tRNA or snRNA substrates [4]. These phenomena raise an intriguing hypothesis that, by mimicking sequence/structural features of noncoding RNAs, mRNAs co-opt those writer proteins to form modified nucleotides, although whether these modifications in mRNAs have become functionally adapted is still unknown.

RNA m^5^C is one of the longest-known RNA modifications. The presence and functions of m^5^C in noncoding RNAs, such as tRNA and rRNA, have been extensively investigated [5–11]. m^5^C has been found recently in mRNAs [12–14] and is suggested to affect most post-transcriptional steps in gene expression [13,15–17]. Functionally, mRNA m^5^C has been shown to impact various biological events [18–21], such as embryonic development, myelopoiesis and cancer cell proliferation.

With the development of an experimental and computational framework to accurately identify mRNA m^5^C sites genome-wide, two types of mRNA m^5^C sites were found in animals [17]. Type I m^5^C sites are adjacent to a downstream G-rich triplet motif and are predicted to be located at the 5^′^ end of stem-loop structures. Interestingly, NSUN2, a tRNA m^5^C methyltransferase, is responsible for Type I mRNA m^5^C methylation [12,13], and the sequence and structural features of Type I mRNA substrates are similar to those of NSUN2’s tRNA substrates. Type II m^5^C sites are adjacent to a downstream UCCA motif and are predicted to be located in loops of stem-loop structures. However, the biogenesis of Type II m^5^C sites, as well as the mechanisms responsible for their selective methylation, remain elusive.

## RESULTS

### Computational inference of a Type II m^5^C site-specific methyltransferase

**Figure 1.**
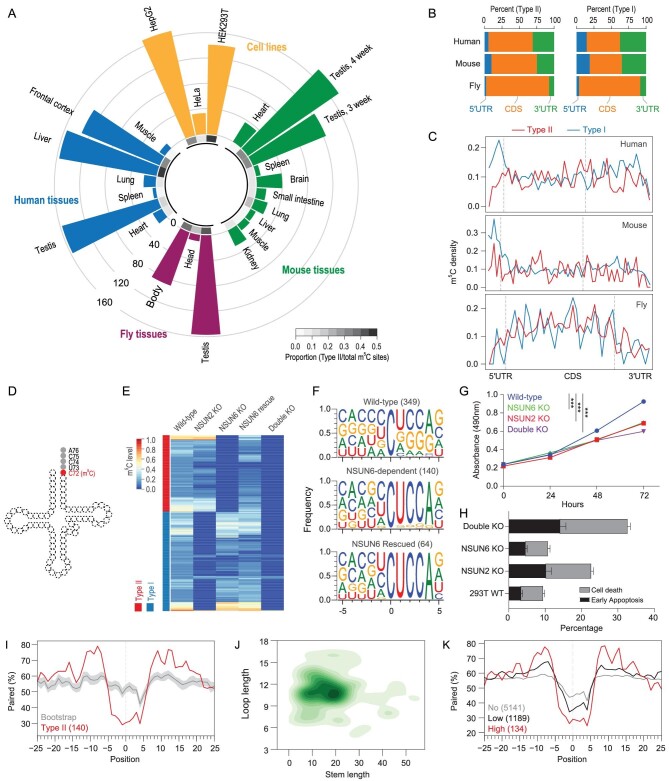
Identification of NSUN6 as an mRNA m^5^C methyltransferase. (A) Circular bar plot showing the proportion and number of Type II m^5^C sites in cell lines and tissues. Inner circle, the proportion of Type II m^5^C sites in each sample; outer circle, the number of Type II m^5^C sites in each sample. The proportion means the number of Type II m^5^C sites divided by the total number of m^5^C sites in a sample. Seven human and 10 mouse mRNA BS-seq samples from our previous study [17] and three wild-type human cell lines and three fly tissues mRNA BS-seq samples generated from this study (Supplementary Table S2) were analyzed. (B) The genic locations of Type II and Type I m^5^C sites in human, mouse and fly. (C) The distributions of Type II and Type I m^5^C sites across the transcripts. The details of the density calculation are described in Methods. (D) Illustration of the tRNA m^5^C site methylated by NSUN6. (E) Heatmap showing the m^5^C methylation levels of sites measured from wild-type, NSUN2 knockout, NSUN6 knockout, NSUN6 rescue and NSUN6/NSUN2 double knockout HEK293T cells. Sites covered by at least 20 reads in all samples and with methylation level ≥0.1 in wild-type cells are shown. (F) The sequence context flanking different groups of sites. The number of sites used for analysis in each group is given in parentheses. NSUN6-dependent, sites that are covered in both wild-type and NSUN6 knockout cells and not methylated (<0.05) in knockout cells; NSUN6 rescued, sites that are covered in both NSUN6 knockout and rescue cells, not methylated (<0.05) in knockout cells, and methylated (≥0.05) in rescue cells. (G) The MTS assay was used to quantify the viable cells in different time points. Data are presented as mean ± SEM (*n* = 4). The *P* values were determined using the Student's t-test by comparing each of the mutant samples with the wild-type samples at 72 hours. ^***^, *P* < 0.001. (H) Propidium iodide (PI) and Annexin V were used to determine the death or early apoptosis of the cells. The percentage of viability was measured at 48 hours. Data are presented as mean ± SEM (*n* = 3). (I) Metaprofiles of the secondary structure of NSUN6-dependent m^5^C sites and flanking regions. Position 0 represents the m^5^C sites. Each negative or positive value indicates the distance between an upstream or downstream position and the m^5^C site. The upstream and downstream 50 nt sequences of the m^5^C sites were extracted from the transcriptome and folded with the RNAfold tool, and the 25 nt flanking regions are shown. The same set of NSUN6-dependent sites as in (F) were used for analysis. The same number of Cs with a UCCA motif on m^5^C-containing genes were sampled 100 times (see Methods), and the median and the quartiles estimated by bootstrap are shown in gray. (J) 2D KDE plot showing the distribution of the stem length and loop length of predicted secondary structures of NSUN6-dependent sites and flanking regions. The same set of NSUN6-dependent sites as in (F) were used for analysis. (K) Metaprofiles of the secondary structure of the NSUN6 miCLIP binding sites and flanking regions. Binding sites with a CUCCA sequence were divided into three groups for analysis: no methylation, <1%;  low-methylation, 1%–10%; and high-methylation, ≥10%.

To identify the potential methyltransferase responsible for Type II m^5^C site methylation, we considered deducing the candidate based on the correlation between Type II m^5^C site number and gene expression level. We first confirmed the UCCA motif as a robust signature of Type II m^5^C sites with the low false-assignment rate of Type I m^5^C sites (Supplementary Note S1, Fig. S1A and B) and then used it to define and characterize Type II m^5^C sites across human, mouse and fly samples profiled with mRNA BS-seq (Supplementary Table S1). The number of Type II m^5^C sites, as well as their proportions (the number of Type II m^5^C sites divided by the total number of m^5^C sites in a sample), varied across samples, ranging from 5 to 169 sites and from 1% to 40% of the total m^5^C sites (Fig. 1A and Supplementary Table S2). Additionally, the distributions of the methylation levels of Type II m^5^C sites also differed among samples (Supplementary Fig. S2A). As exemplified by several tissues or cells with relatively high numbers of Type II m^5^C sites, the methylation levels of top sites typically ranged from 40% to 90% (Supplementary Fig. S2B). Interestingly, both conserved and lineage-specific methylation profiles of Type II m^5^C sites were observed. For example, Type II m^5^C sites were highly enriched in testis in all three species studied, and enriched only in human liver but not in mouse liver. Moreover, Type II and Type I m^5^C sites had an overall similar distribution of genic locations (Fig. 1B), although compared with Type I m^5^C sites, a slightly lower proportion of Type II m^5^C sites was found in 5^′^UTR regions in human and mouse (Fig. 1C, Supplementary Table S3).

Next, we examined the correlation between the expression levels of each of 125 known methyltransferases and the number of Type II m^5^C sites across nine human samples. Among them, we identified NSUN6, which was originally identified as a tRNA methyltransferase, as the one with the highest correlation coefficient (Supplementary Fig. S2C, Table S4). A strong positive correlation was also observed in mouse samples (Supplementary Fig. S2D). Interestingly, NSUN6 is known to specifically target the C72 position at the 3^′^end of tRNA^Thr^ and tRNA^Cys^ with a UCCA tail in humans (Fig. 1D) [22,23], in line with the enriched UCCA motif of Type II m^5^C sites. Taken together, these results enabled us to predict that NSUN6 is a Type II m^5^C site-specific methyltransferase.

### Experimental verification of NSUN6 as the methyltransferase responsible for Type II m^5^C sites

To experimentally verify our prediction, we generated NSUN6 knockout human cells (Supplementary Fig. S3A, B and Table S5). Human HEK293T cells were selected because of the high proportion of Type II m^5^C sites observed. We found that 49.8% (140/281) of m^5^C sites identified in the wild-type sample were not methylated in the knockout sample (Fig. 1E, Supplementary Note S2), and the methylation of these sites was partially rescued by transiently expressing NSUN6 in the knockout cells (Fig. 1E). A UCCA motif was found to be adjacent to most (123/140) NSUN6-dependent sites (Fig. 1F), and the loss of methylation on sites with a UCCA motif was further confirmed using NSUN6 knockout HeLa cells (Supplementary Fig. S3C). Notably, compared with HEK293T cells, HeLa cells had a lower NSUN6 expression level (Supplementary Fig. S3D). Only 30 sites were identified as NSUN6-dependent Type II m^5^C sites in HeLa cells; among them, 27 sites were overlapped with Type II m^5^C sites in HEK293T cells. When both NSUN6 and NSUN2 were knocked out in HEK293T cells (Supplementary Fig. S3A and B), nearly all mRNA m^5^C was eliminated (Fig. 1E). These observations indicate that NSUN6 is an mRNA methyltransferase and, together with NSUN2, determines most mRNA m^5^C.

Characterization of NSUN6 knockout HEK293T cells revealed high viability but reduced proliferation (Fig. 1G, H and Supplementary Fig. S3E). Interestingly, no proliferation defect was observed for NSUN6 knockout HeLa cells (Supplementary Fig. S3F), which may be because NSUN6 contributed little to mRNA m^5^C in HeLa cells (Fig. 1A and Supplementary Fig. S3C). It should be noted that we cannot exclude the possibility that the proliferation defects may result from the lack of tRNA methylation. As a comparison, we examined NSUN2 knockout HEK293T cells and revealed reduced cell viability (Fig. 1G and H), consistent with the previous studies [24]. Finally, we found that NSUN6/NSUN2 double knockout HEK293T cells exhibited a more severe cell proliferation defect as compared with the single knockout cells (Fig. 1G and H). These findings highlight the possible physiological importance of NSUN6.

Next, we examined the structural feature of NSUN6-dependent sites. Compared with background cytosines (see Methods), NSUN6-dependent sites had a lower percentage of base-pairing in the ∼5 nt flanking regions and a higher percentage of base-pairing in the 10–15 nt flanking regions (Fig. 1I and J). We then utilized miCLIP data to confirm the NSUN6-mediated methylation of Type II m^5^C sites [7,25]. As expected, frequent reverse transcription stalling was observed at the NSUN6-dependent, but not the NSUN6-independent, sites in NSUN6 miCLIP data, and the opposite pattern was observed in NSUN2 miCLIP data (Supplementary Fig. S3G). Interestingly, about 59% of the NSUN6 binding sites were adjacent to a downstream UCCA motif. Moreover, the methylation status of these UCCA motif sites was positively correlated with the strength of the predicted stem-loop structure (Fig. 1K). These results confirmed the direct binding and sequence context requirement for NSUN6-mediated mRNA methylation.

As NSUN6 is found in both vertebrates and invertebrates, we asked whether NSUN6 also methylates Type II m^5^C sites in invertebrates. NSUN6 knockout flies were generated and verified by RT-PCR (Supplementary Fig. S4A and B). We first confirmed that fly NSUN6 also methylated the 3^′^end of tRNA^Thr^ and tRNA^Cys^ (Supplementary Fig. S4C). Next, we examined the mRNA m^5^C methylation. We found that a group of mRNA m^5^C sites, whose sequence and structural features were similar to that of Type II m^5^C sites in mammals, lost their methylation in NSUN6 knockout flies (Supplementary Fig. S4D and E), although their motif had a stronger position −1 C preference and a wider base tolerance at position +3. These results are consistent with the predicted conserved NSUN6-RNA complex structure between vertebrates and invertebrates (Supplementary Fig. S4F), suggesting NSUN6 as a conserved mRNA m^5^C writer.

### Validation of the sequence and structural requirement for NSUN6-mediated mRNA methylation

**Figure 2.**
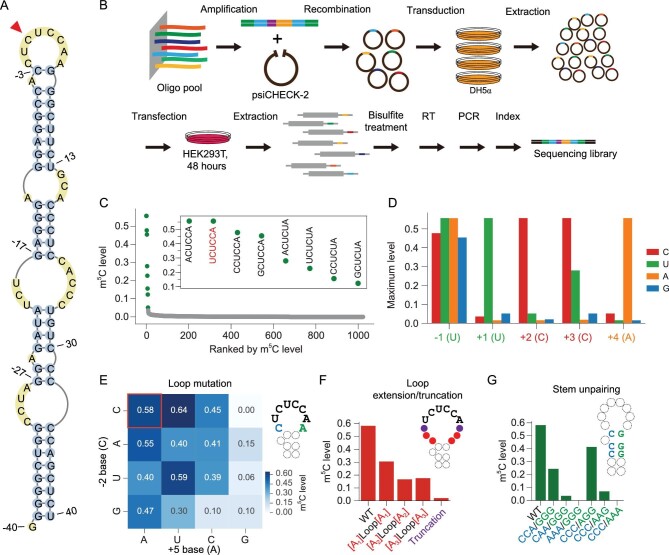
Validation of the sequence and structural requirement for NSUN6-mediated mRNA methylation. (A) The predicted secondary structure of substrate#1 (m^5^C site: chr15:91425541). The m^5^C site is indicated by the red arrowhead. (B) The schema of the methylation reporter assay. (C) The NSUN6 core motif (Nm^5^CUCCA) was systematically mutated and the ranked methylation levels of each variant are shown. The original motif of the substrate is colored in red. Y-axis represents the methylation levels of the substrates with the indicated motif sequences. One targeted BS-seq library was constructed. Only motifs with at least 20 reads covered are shown (Supplementary Table S6). (D) Bar plot showing the maximum methylation levels of the substrates with mutations in the core motif. Y-axis represents the maximum methylation levels of each of the four bases in each position. The maximum level is represented using the methylation level of the substrate with the highest methylation level with the indicated base. (E) Heatmap showing the methylation levels of −2 and/or +5 base substitutions. The original bases are highlighted in red. The number of reads covered for each sequence are shown in Supplementary Table S6. (F) The methylation levels of the substrates with truncation or extension of the loop region. Truncation, bases at positions −2 and +5 were deleted from the original loop. A_1_, A_2_, A_3_ and A_4_ mean that one to four As were added up- and downstream of the loop region to extend the loop. Y-axis represents the methylation levels of the substrates with the indicated sequences. The number of reads covered for each sequence is shown in Supplementary Table S6. (G) The methylation levels of the substrates of which the base-pairing of the stem regions were disrupted by replacing C/G by As. Y-axis represents the methylation levels of the substrates with the indicated sequences. The number of reads covered for each sequence is shown in Supplementary Table S6.

To examine the impact of the sequence and structural features identified in our computational model for achieving methylation, we employed a high-throughput mutagenesis assay [26]. Four Type II m^5^C sites with moderate or high methylation levels in HEK293T cells were selected for the assay (Fig. 2A and Supplementary Fig. S5). On the basis of these targets, we designed sequence variants to systematically impact the sequence motif and secondary structure surrounding the m^5^C sites (Supplementary Table S6). Each of these sequences further comprised a barcode and common adapters on both ends. The sequences were synthesized as DNA oligo pools, cloned as 3^′^UTR elements downstream of a luciferase gene, and transfected into wild-type HEK293T cells. Their methylation levels were determined by targeted BS-seq (Fig. 2B).

We began by measuring the extent to which each individual nucleotide surrounding the m^5^C site was required for methylation. Overall, except for the original sequence, only several other sequence variants were highly methylated (Fig. 2C and Supplementary Fig. S5). More specifically, we found that 1) position +3 favored C and U, and 2) original base composition at positions +1, +2 and +4 led to better methylation (Fig. 2D and Supplementary Fig. S5). We next examined the impact of the more distal flanking bases. For positions that are adjacent to the m^5^C core motif, methylation was more sensitive to position +5 A to G/C replacement, position −3 A to C/U replacement but less sensitive to position −2 mutations (Fig. 2E and Supplementary Fig. S5). Other efforts were made in modifying the loop and the stem. We found that both the loop and the stem lengths tuned the methylation levels (Fig. 2F, G and Supplementary Fig. S5). These results together suggest that both the sequence motif and the stem-loop structure are important for the methylation process.

### Characterizing the NSUN6-mRNA interaction

To further reveal the mechanisms responsible for the sequence- and structure-selective recognition and methylation of mRNA substrates, we sought to use Rosetta comparative modeling [27], reporter assay and *RNP-denovo* [28] to model NSUN6-RNA complex structures in multiple species and identify residues responsible for NSUN6-RNA interaction.

**Figure 3.**
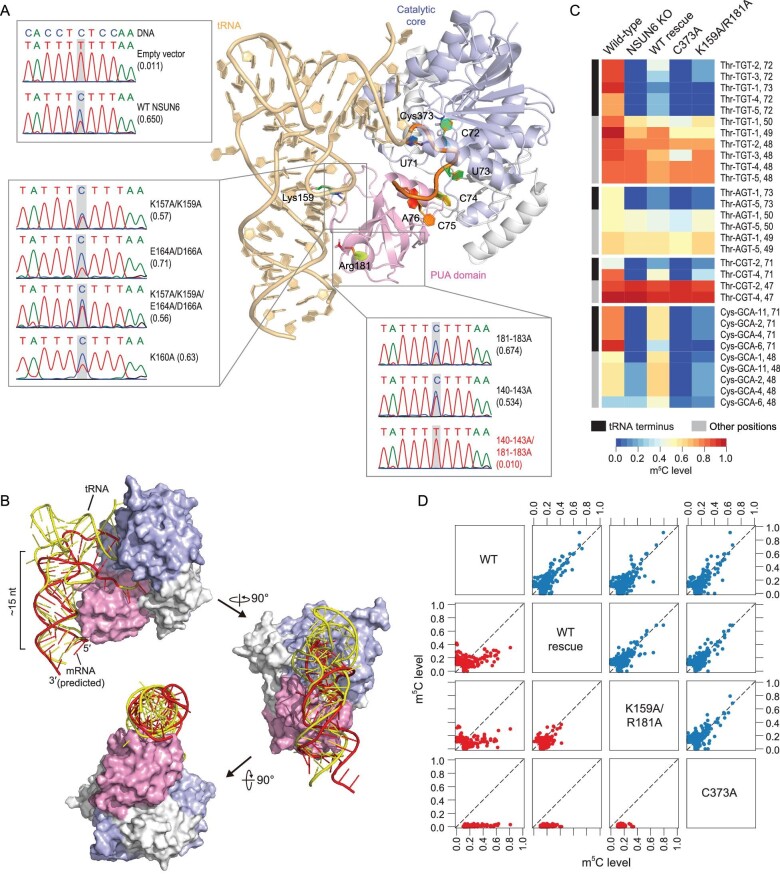
Identification of an NSUN6 variant that largely loses tRNA methylation but retains mRNA methylation ability. (A) The overall structure of the human NSUN6-tRNA complex (PDB: 5wws) [29]. The Um^5^CUCCA fragment, MTase domain, PUA domain and key residues are highlighted. The Sanger sequencing traces for the PCR amplicons of the methylation reporter targeted by different NSUN6 variants are also shown. 140–143A/181–183A variant that led to a loss of methylation is highlighted in red. (B) A proposed NSUN6-mRNA structural model in a tRNA-like mode, along with NSUN6-tRNA co-crystallization structure (PDB: 5wws). tRNA is colored in yellow and mRNA is colored in red. (C) Heatmap showing the methylation profiles of isodecoders of tRNA^Thr^ and tRNA^Cys^ in the wild-type cells, NSUN6 knockout cells, and NSUN6 knockout cells rescued by wild-type NSUN6 or NSUN6 variants. Sites covered by at least 10 reads in all samples and with a methylation level of ≥0.1 in the wild-type cells are shown. (D) Pairwise comparison of mRNA methylation levels between the wild-type cells and NSUN6 knockout cells rescued by wild-type NSUN6 or NSUN6 variants. Type I and Type II m^5^C sites are shown in the lower panel (red) and upper panel (blue), respectively. For each pair, sites covered by at least 20 reads in both samples and with a methylation level of ≥0.1 in at least one sample are shown.

Based on the known human NSUN6-tRNA co-crystallization data (Fig. 3A) [29], we first modeled the NSUN6-tRNA structures in mouse and fly and examined the residues that were predicted to interact with the core m^5^C motif, particularly positions −1 and +3 of m^5^C sites, which differ between vertebrates (human and mouse) and invertebrates (fly) (Supplementary Fig. S6A and B). We found that, compared with human and mouse NSUN6, fly NSUN6 had a more negatively charged methylation pocket (Supplementary Fig. S6C and D), which might lead to the stronger C base preference of position −1 in fly. A previous study showed that position +3 (tRNA C75) interacted with the methylation pocket residues, including Tyr131, Lys192, Gly193 and Asp209 [29]. In human and mouse, U was less favored at position +3 because the reduced hydrogen bond might form between U and NSUN6, while A or G at position +3 was not allowed because of steric hindrance (Supplementary Fig. S6E and F). In the fly, all four bases might be allowed at position +3 because Asp209 and Lys192 were replaced by Gly and Ser, which resulted in a larger space for position +3 bases (Supplementary Fig. S6E and F); C remained the most favorable base because it might have more interactions with the residues (Supplementary Fig. S6F).

Next, we examined the interaction between NSUN6 and mRNA stem-loop structure. Given that the PUA domain is responsible for D-stem recognition in tRNA methylation [29] and mRNA substrates are predicted to have a stem-loop structure, we predicted that NSUN6 would also interact with mRNA substrates via the PUA domain. Based on the NSUN6-RNA structural model, we performed mutagenesis that might lead to significant shape/electronic changes on the PUA domain (Supplementary Fig. S6G and H) and used a Type II m^5^C site reporter (see Method) to examine the methylation activity of these NSUN6 variants. We found that 140–143A/181–183A variant, which severely breaks the positively charged distal end surface of the PUA domain, led to a loss of methylation (Fig. 3A), while other variants retained mRNA methylation activity (Fig. 3A). These results suggest that, unlike NSUN6-mediated tRNA methylation that is sensitive to single residue mutation [29], mRNA m^5^C is PUA-dependent but less sensitive to specific residues.

Last, we constructed the NSUN6-mRNA complex structure by folding and docking mRNA stem to the NSUN6 protein surface using *RNP-denovo* (Methods) and attempted to reveal the possible difference between NSUN6-tRNA and NSUN6-mRNA interactions. Because most NSUN6 mRNA substrates had a similar stem-loop structure (Supplementary Fig. S7A), we used the four substrates examined in the high-throughput mutagenesis assay for modeling. We found that one of the best-scored models fitted our current understanding of NSUN6-mRNA recognition: mRNA in a tRNA-like conformation (Fig. 3B and Supplementary Fig. S7B). In this model, the mRNA strand twisted as the tRNA anticodon stem, forming a ∼15 nt stem whose axis overlapped with that of anticodon stem. This model explained why mutations around residues 157–163 retained mRNA methylation activity: although these residues (e.g. Lys159) might be essential for tRNA methylation, they were away from the mRNA strand in the NSUN6-mRNA model. Notably, accurate prediction and modeling of protein-RNA structure is a major challenge for RNA binding proteins, hence, the generated model needs to be verified in future studies.

### Identification of an NSUN6 variant that largely loses tRNA methylation but retains mRNA methylation ability

Results from the reporter assay and NSUN6-mRNA structure modeling suggest that tRNA and mRNA substrates may interact with different residues of NSUN6, thus tRNA methylation and mRNA methylation may be separated by modifying residues in NSUN6. As proof of concept, we selected the known tRNA methylation null mutant K159A/R181A [29] for a test. Given that residues 181–183 do not affect mRNA methylation, we predicted that this mutant may not affect mRNA methylation.

To verify our prediction, we transiently expressed the K159A/R181A mutant in NSUN6 knockout HEK293T cells, using the catalytically inactive C373A mutant and wild-type NSUN6 as the negative and positive controls, respectively, and then conducted mRNA and tRNA m^5^C profiling. For tRNA, as expected, C72 of tRNA^Thr-TGT^, tRNA^Thr-CGT^, tRNA^Thr-AGT^ and tRNA^Cys-GCA^ completely lost their methylation in both the knockout cells and the C373A mutant rescued cells (Fig. 3C, Supplementary Fig. S8A, B and Note S3). Compared with the cells rescued by wild-type NSUN6, very low levels of methylation were found in the K159A/R181A mutant (Fig. 3C and Supplementary Note S3). For mRNA, Type II m^5^C sites lost all methylation in both the knockout cells and the C373A mutant rescued cells, but similar Type II m^5^C site methylation profiles were observed between the K159A/R181A rescued cells and the wild-type NSUN6 rescued cells (Fig. 3D). These findings suggest that tRNA methylation and mRNA methylation may be distinguished via use of an edited NSUN6 protein.

### Methylation status of Type II and Type I mRNA m^5^C sites in different cellular compartments

**Figure 4.**
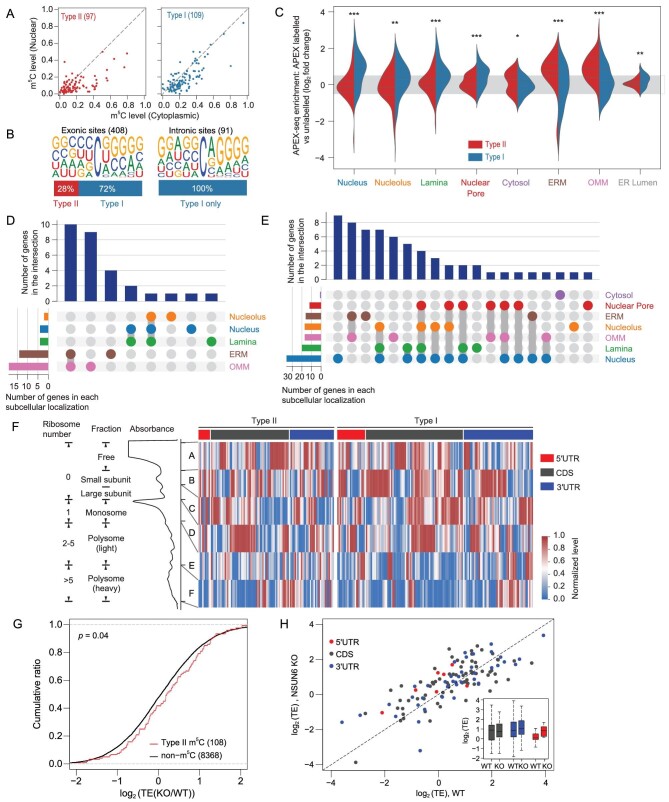
Characterizing the deposition and the molecular readout of Type II and Type I m^5^C sites. (A) Comparison of methylation levels of m^5^C sites measured in nuclear and cytoplasmic poly(A)+ RNAs. Sites covered by at least 20 reads in both samples are shown. (B) Motif analysis for m^5^C sites called from nuclear poly(A)+ RNAs. Exonic sites and intronic sites were analyzed separately. The percentages of Type I and Type II m^5^C sites in exonic and intronic sites are also shown. (C) Violin plot showing the enrichment of genes containing Type I or Type II m^5^C sites in different subcellular localizations. HEK293T APEX-seq data were used for analysis [30]. Y-axis represents the APEX-seq enrichment, which means log2fold change over the corresponding negative controls. Inner bars, median and two quartiles. The significant difference between genes containing Type II and Type I m^5^C sites was determined using a two-sided Mann–Whitney U test. ^*^, *P* < 0.05; ^*^^*^, *P* < 0.01; ^*^^*^^*^, *P* < 0.001. (D and E) UpSet plot showing the overlapping number of genes with Type II (D) or Type I (E) m^5^C sites in different subcellular localizations. UpSet plots the intersections of a set as a matrix. Each row corresponds to a set (subcellular localization). Cells are either empty (light-gray), indicating that this set is not part of that intersection, or filled, showing that the set is participating in the intersection. Therefore, the bar plot on the top shows the number of genes in the intersection; the bar plot on the bottom shows the number of genes in each subcellular localization. (F) Methylation levels of sites across the polysomal fractions. Absorbance trace (260 nm) across the gradients processed for RNA BS-seq is shown above the heatmap. Each row represents a Type I or Type II m^5^C site. 154 and 106 Type I and Type II sites with at least 20 reads in at least five fractions are shown. The data were Min-Max normalized. In brief, for each site, the original methylation levels were normalized to interval [0,1], according to the following equation: ${L_{\rm norm}} = \frac{{L - {L_{\rm min}}}}{{{L_{\rm max}} - {L_{\rm min}}}}$, where *L* is the original value, *L*_min_ is the minimal value of the original value, *L*_max_ is the maximal value of the original value and *L*_norm_ is the normalized value. (G) Comparison of TEs between Type II m^5^C and non-m^5^C genes in wild-type and NSUN6 knockout HEK293T cells. m^5^C genes were defined as genes containing methylated Type II m^5^C sites (methylation level > 0.1) in HEK293T cells. Genes with mRNA and RPF abundance (mRNA and RPF RPKM) >2 in both wild-type and knockout cells were analyzed. The *P* value was determined using a one-sided Kolmogorov-Smirnov Test. (H) Comparison of TEs of Type II m^5^C genes between wild-type and NSUN6 knockout HEK293T cells. Genes with m^5^C sites in different genic locations are indicated by different colors.

Next, we sought to assess within which subcellular compartments Type II and Type I m^5^C sites are localized. We purified nuclear and cytoplasmic-enriched subcellular fractions in HEK293T cells and performed mRNA BS-seq on these fractions. The purity of the nuclear and cytoplasmic fractions was confirmed by quantitative PCR (qPCR) and western blot (Supplementary Fig. S9A–C). We found that m^5^C levels of a substantial fraction of Type II m^5^C sites were higher in the cytoplasm than in the nucleus, while most Type I m^5^C sites had similar m^5^C levels between the two subcompartments (Fig. 4A). Moreover, when examining the m^5^C sites called from nuclear RNA, only Type I m^5^C sites were observed in introns (Fig. 4B). At the protein level, in HEK293T cells, NSUN2 was mainly located in the cytoplasm; in HeLa and HepG2 cells, NSUN2 was located in both the nucleus and cytoplasm (Supplementary Fig. S9C). The majority of NSUN6 was located in the cytoplasm (Supplementary Fig. S9C), consistent with the known subcellular location of NSUN6 in the Golgi apparatus [22]. In line with this, NSUN6 miCLIP data revealed that more than 89.8% of the NSUN6 binding sites were located in exonic regions.

### Potential function and molecular readout of Type II m^5^C methylation

A recent development, the APEX-seq, is a method for direct proximity labeling of RNA using the peroxidase enzyme APEX2, enabling study of the enrichment of mRNA species in different subcellular localizations [30]. We used published APEX-seq data in nine distinct subcellular localizations of HEK293T cells to examine the subcellular localizations of m^5^C-containing transcripts. We found that transcripts containing Type I m^5^C sites were mainly enriched in the nuclear fractions (nucleus, nucleolus, lamina and nuclear pore) (Fig. 4C–E). Transcripts containing Type II m^5^C sites were mostly enriched near the endoplasmic reticulum membrane (ERM) and outer mitochondrial membrane (OMM), but not in the endoplasmic reticulum lumen (Fig. 4C–E).

As a negative correlation between translational efficiency and the presence of mRNA m^5^C has been reported previously [17], we asked whether mRNA m^5^C plays a role in translational control. First, we fractionated the lysate of wild-type HEK293T cells to isolate mRNAs associated with different numbers of ribosomes, and performed mRNA BS-seq in each fraction. A comparison of each fraction revealed that the highest methylation levels of m^5^C sites were more frequently found in mRNAs not bound by the ribosome, and the lowest methylation levels were more frequently found in mRNAs bound by multiple ribosomes (Fig. 4F and Supplementary Fig. S10A). Next, we generated ribosome profiling data for wild-type and NSUN6 knockout HEK293T cells. Compared with non-m^5^C genes, genes containing Type II sites had an overall slightly increased translation efficiency (TE) upon NSUN6 knockout (Fig. 4G). When examining Type II m^5^C genes individually, NSUN6 knockout led to both increased and decreased TEs, with the effects varying dependent on the genic locations (Fig. 4H). Last, to further measure the impact of m^5^C methylation on translational control, we selected three Type II m^5^C sites (two in CDS and one in 3^′^UTR) with high-methylation in HEK293T cells for a dual-luciferase reporter assay. We cloned individual m^5^C sites and flanking regions into the reporter gene and generated C-to-T point-mutation as a control (Supplementary Table S7, Methods). We first confirmed that the wild-type plasmids were methylated when expressed in HEK293T cells (Supplementary Fig. S10B). Next, each of the wild-type or mutated plasmids was transfected into the wild-type or NSUN6 knockout HEK293T cells. We found that methylated transcripts had a slightly decreased luciferase level (Supplementary Fig. S10C, D and Table S8). We also examined the protein expression of genes with selected m^5^C sites in the wild-type and NSUN6 knockout cells. We found that one gene tended to have a decreased protein expression in the wild-type cells, although it had a slightly increased RNA level in the wild-type cells (Supplementary Fig. S10E and F). These results together suggest that, although an overall weak negative correlation between mRNA m^5^C and translational efficiency is observed, the effects of individual sites may vary depending on the genic locations.

## DISCUSSION

In this study, we identified NSUN6 as an mRNA methyltransferase. We found that NSUN6 specifically methylated a group of sites in a sequence- and structure-selective manner. Genes containing Type II m^5^C sites were enriched near the outer mitochondrial membrane, suggesting that NSUN6-mediated methylation might be involved in mitochondrial function. Notably, NSUN6 showed a tissue-specific expression pattern, with higher expression levels in testis, pituitary, ovary, liver and thyroid (https://www.proteinatlas.org/ENSG00000241058-NSUN6/tissue). Consistently, more Type II m^5^C sites were observed in tissues with high-level NSUN6 expression (Fig. 1A). Our findings pave the path toward mechanistic dissection of the physiological importance of NSUN6, such as its roles in cell proliferation and tumor progression [31,32].

NSUN6 is a methyltransferase known to methylate C72 of tRNA^Thr^ and tRNA^Cys^. Compared with its tRNA methylation sites, most of NSUN6’s mRNA sites had much lower methylation levels. For example, of the 503 Type II sites found in at least one of the 10 human tissue and cell samples (Fig. 1A), only 61 sites had a methylation level > 0.4 in one or more samples. Interestingly, it is known that archaeal NSUN6 has a much wider range of tRNA substrates [33], but both human and fly NSUN6 proteins only methylated four tRNAs at C72. Moreover, miCLIP data revealed that about 30% of human NSUN6 miCLIP reads were mapped to mRNAs. These data suggest that NSUN6 in higher animals may be evolving to bind fewer tRNAs but more mRNAs, although many of the mRNA substrates are still methylated at low levels.

One of the most challenging problems in the RNA modification field is that most RNA modification writer proteins have multiple types of RNA substrates, thus it is difficult to study the specific functions of one type of RNA substrate. Here using NSUN6 as a model, based on protein-RNA structure analysis, we were able to identify an NSUN6 variant that largely loses tRNA methylation but retains mRNA methylation ability. We anticipate that our approach is applicable to other writer proteins, such as NSUN2 (m^5^C), METTL16 (m^6^A), TRMT6/TRMT61A (m^1^A) and PUS proteins (pseudouridine). Similar to NSUN6, although their mRNA substrates mimic the features of tRNA or snRNA [4], they are likely to have more flexible structural or sequence features and their methylation may require a less stringent protein-RNA interaction. Therefore, it is possible to generate variants that lose tRNA or snRNA modification but retain mRNA modification ability, and these variants may enable the specific investigations of the functions of their mRNA substrates.

## METHODS

Detailed descriptions of methods are available as Supplementary data.

## DATA AND CODE AVAILABILITY

All sequencing data are available in the GEO database under accession number GSE148764.

## Supplementary Material

nwaa273_Supplemental_File
