# Supplementary material for: Sequence- and structure-selective mRNA m5C methylation by NSUN6 in animals
Source: Natl Sci Rev. 2020 Oct 31;8(6):nwaa273. doi: 10.1093/nsr/nwaa273 (PMC8288183; doi:10.1093/nsr/nwaa273)
Supplement: nwaa273_Supplemental_File [file nwaa273_supplemental_file.zip › NSUN6 supplementary_materials_NSR_formatted.docx]

**Supplementary Information**

**Supplementary Figures**


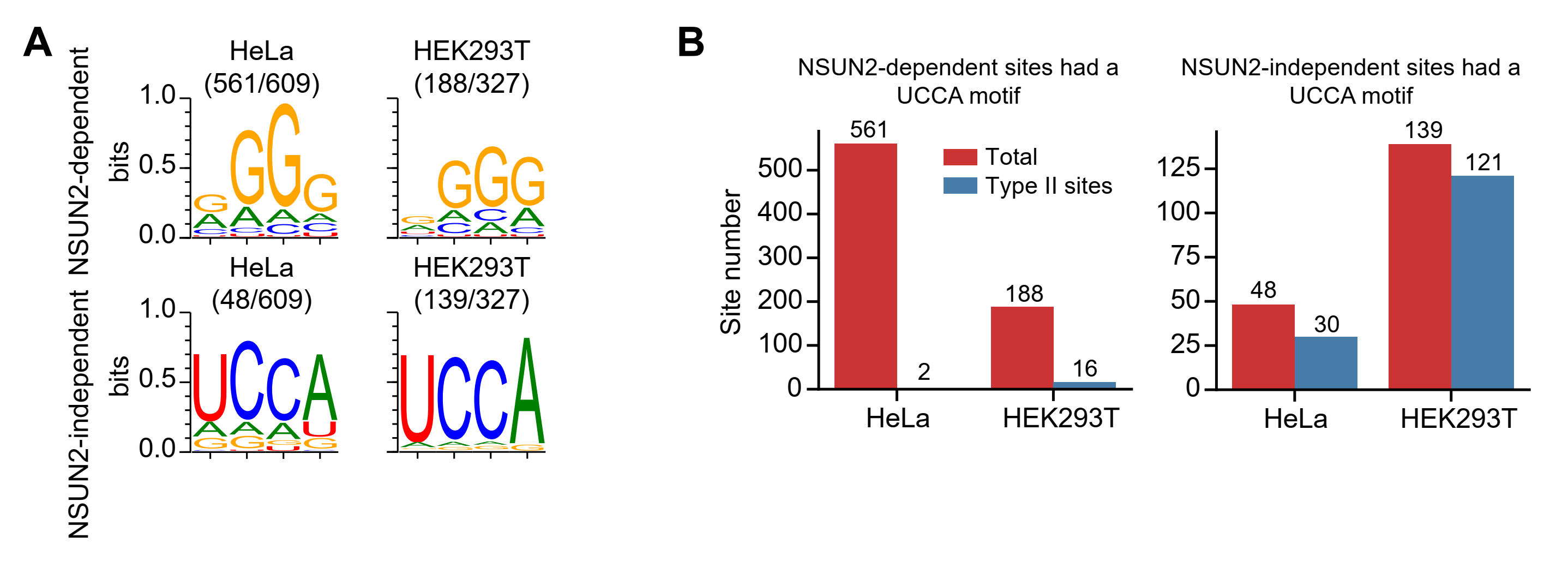


**Figure S1. Analysis of NSUN2-dependent and -independent sites.**

(**A**) The sequence motif surrounding NSUN2-dependent and -independent sites called from HeLa and HEK293T cells. NSUN2-dependent sites, m^5^C sites identified in wild-type cells but not methylated (m^5^C level < 5%) in NSUN2 knockout cells; NSUN2-independent sites, m^5^C sites methylated in both wild-type and NSUN2 knockout cells. HeLa cell data were from a previous study [[1](#_ENREF_1)]. HEK293T cell data were generated in this study. The proportion of sites is given in parenthesis. The motifs were generated using WebLogo 3.

(**B**) The numbers of NSUN2-dependent or -independent sites that are with a UCCA motif in HeLa and HEK293T cells.


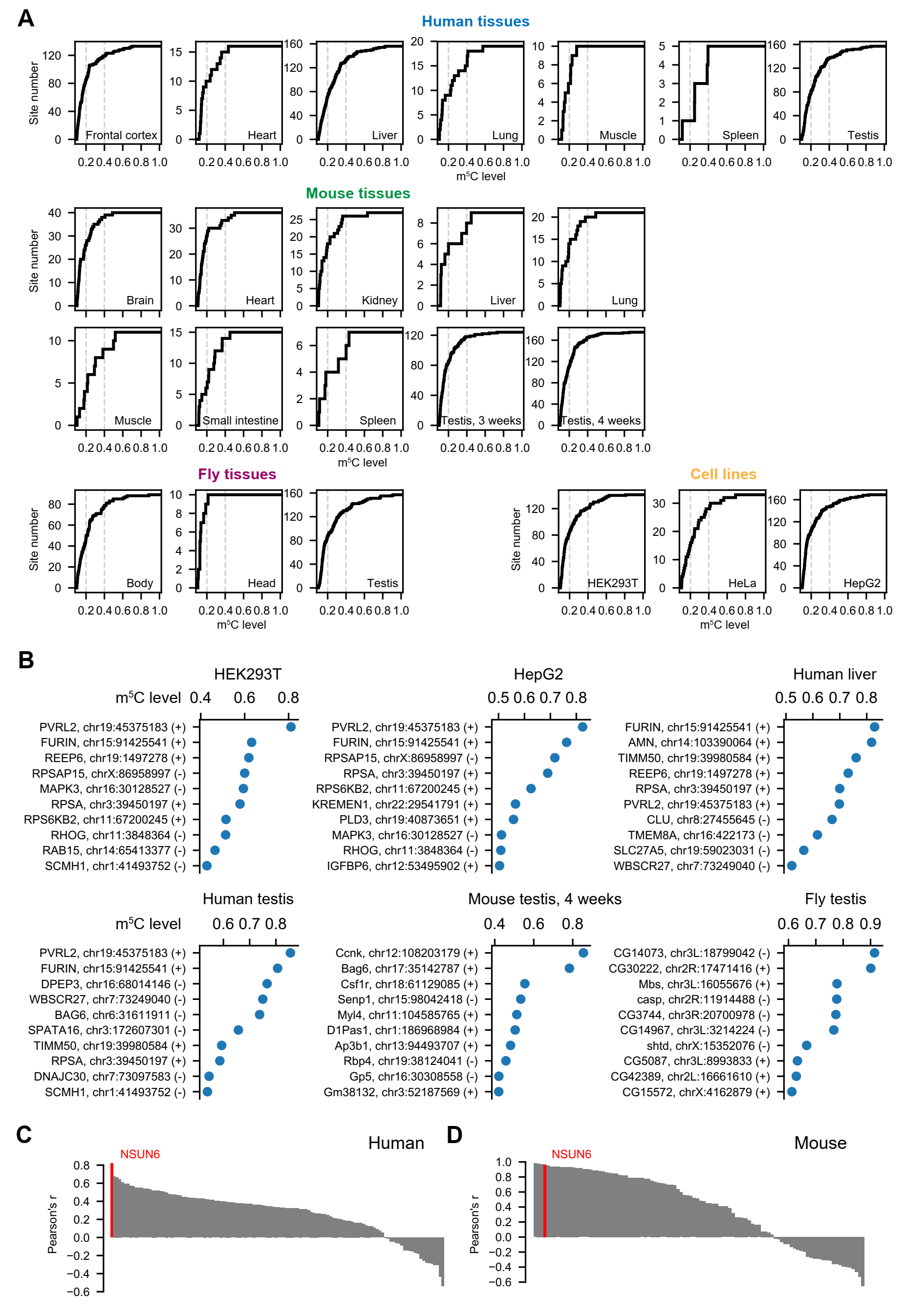


**Figure S2. Computational inference of a Type II m^5^C site-specific methyltransferase.**

(**A**) The cumulative curve of the number of m^5^C sites with different methylation levels. Samples in **Figure 1A** were analyzed.

(**B**) Top 10 methylated Type II sites in selected tissue or cells.

(**C-D**) Barplot showing the correlation between the expression levels of all possible methyltransferases and the number of Type II m^5^C sites in human (**C**) or mouse samples (**D**). Human and mouse gene expression data were from GTEx and modENCODE projects, respectively.


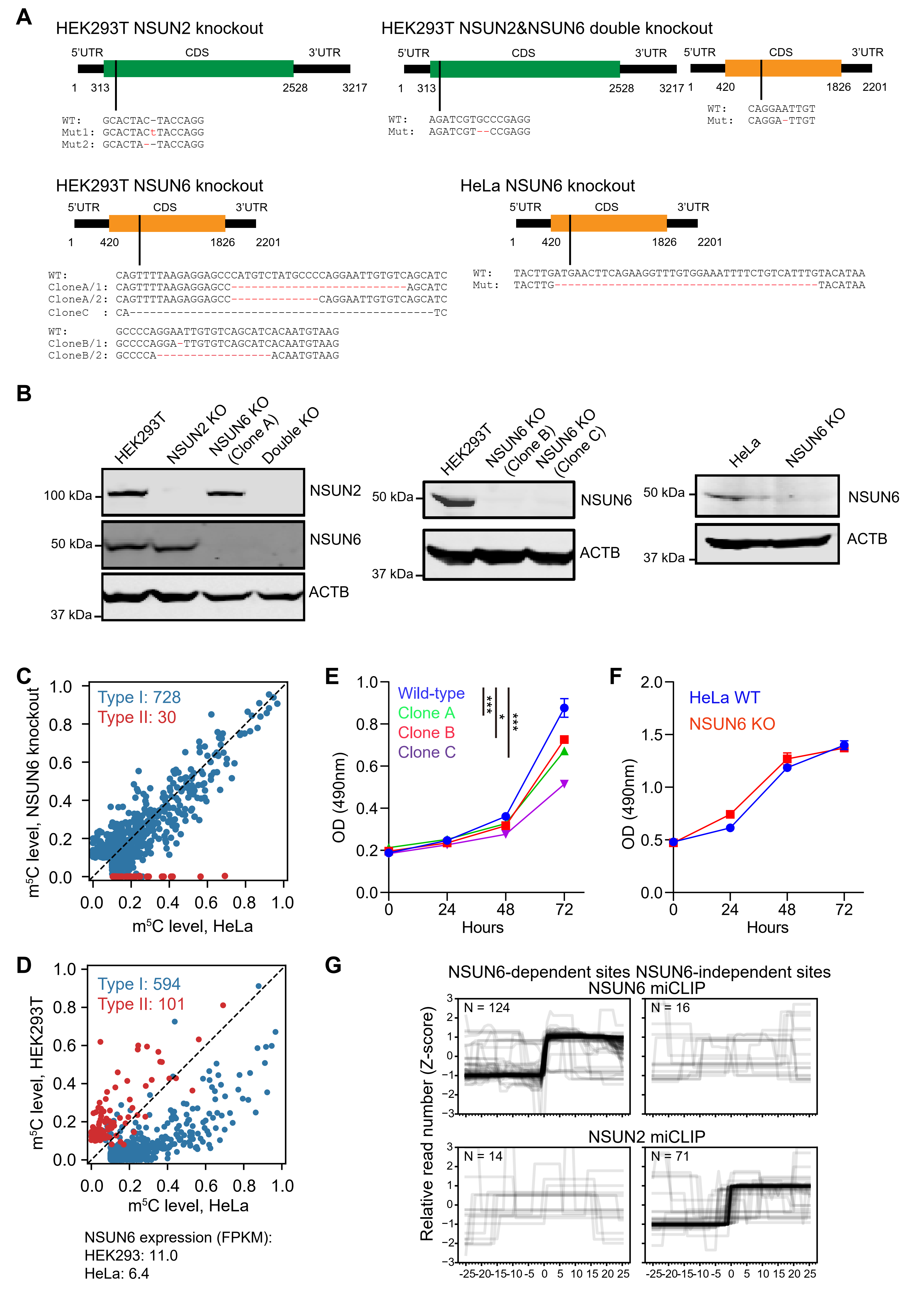


**Figure S3. Knockout cell generation and the analysis of miCLIP data.**

(**A**) Schematic representation of knockout generation using the CRISPR/Cas9 system. The sequence information for the mutants is indicated. All mutant alleles result in a premature stop codon. For NSUN6 knockout HEK293T cells, we generated three independent clones (Clone A, B, and C). Clone A was used for all the experiments; Clone B and C were only used for the experiment in **Figure S3B&E**.

(**B**) Western blot validation of the mutagenesis.

(**C**) Comparison of methylation levels of m^5^C sites measured between wild-type and NSUN6 knockout HeLa cells. The numbers of Type II and Type I m^5^C sites are indicated. Sites covered by at least 20 reads in both samples and with a methylation level of ≥ 0.1 in either wild-type or NSUN6 knockout HeLa cells are shown.

(**D**) Comparison of methylation levels of m^5^C sites measured between HEK293T and HeLa cells. The numbers of Type II and Type I m^5^C sites are indicated. Sites covered by at least 20 reads in both samples and with a methylation level of ≥ 0.1 in either HEK293T or HeLa cells are shown.

(**E**) The MTS assay was used to quantify viable cells at different time points. Data are presented as Mean ± SEM (n = 4). The p values were determined using the Student's t-test by comparing each of the mutant samples with the wild-type samples at 72 hours. *, p < 0.05; ***, p < 0.001.

(**F**) The MTS assay was used to quantify viable cells at different time points. Data are presented as Mean ± SEM (n = 4).

(**G**) The distributions of miCLIP reads around mRNA m^5^C sites. Normalized miCLIP read counts were plotted around the NSUN6 -dependent and -independent sites using NSUN2 and NSUN6 miCLIP data. The upstream and downstream 25 nt regions of the m^5^C sites were selected and read counts were normalized with Z-score. The number of sites (N) that can be covered by miCLIP reads is indicated.


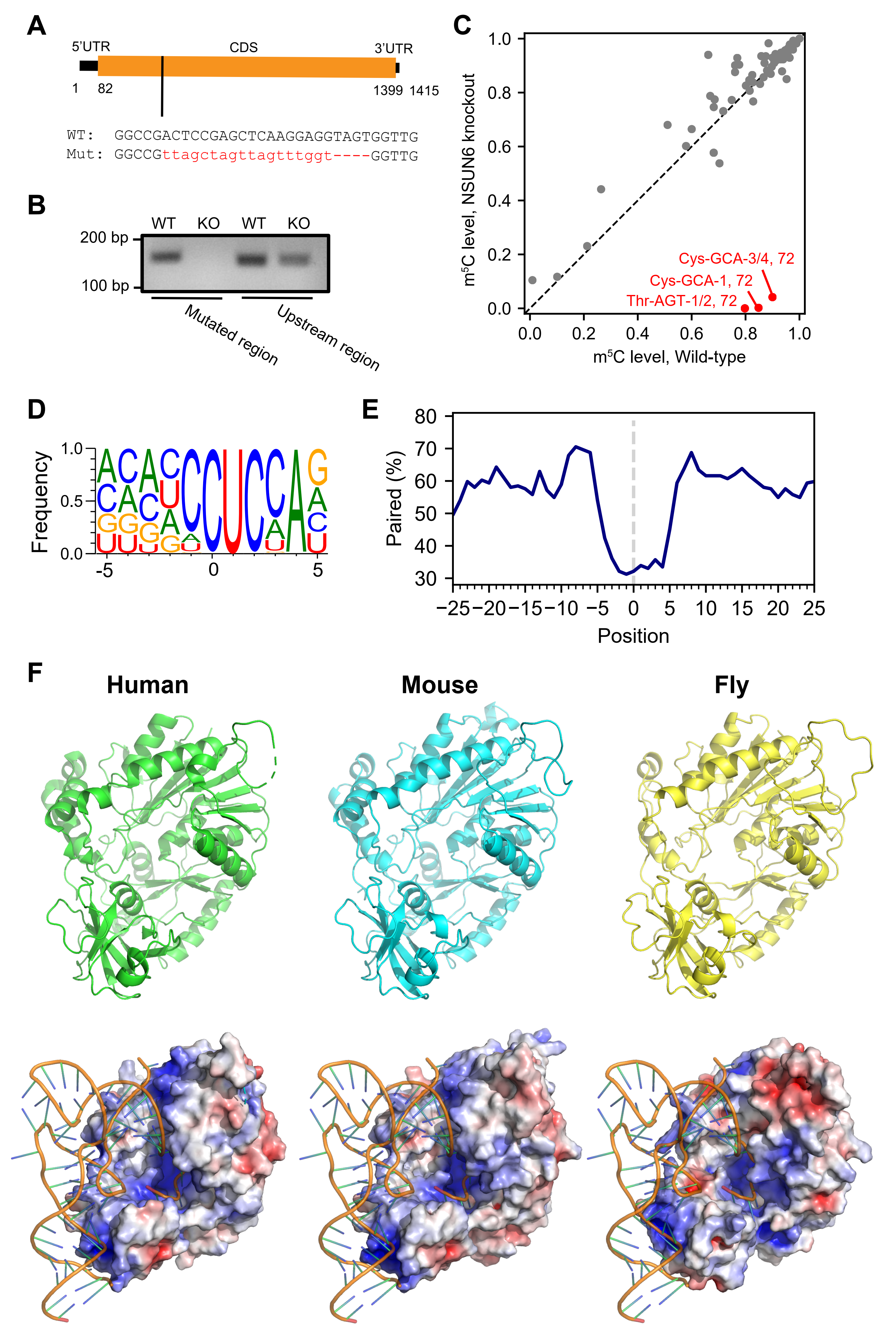


**Figure S4. NSUN6 methylates Type II m^5^C sites in fly.**

(**A**) Schematic representation of NSUN6 mutant generation using the CRISPR/Cas9 system. A mutant that produced an INDEL at CDS region, leading to a premature stop codon at amino acid position 18 (the short isoform) or 99 (the long isoform), was selected.

(**B**) RT-PCR verification of NSUN6 expression in wild-type and mutant flies. RNA from whole body samples of 5 days old adult male flies were used for analysis. A primer pair was designed to target the mutated region so only wild-type transcript can be amplified; another primer pair was designed to target the upstream of the mutated region so both wild-type and mutated transcripts can be amplified.

(**C**) Comparison of methylation levels of tRNA m^5^C sites between wild-type and NSUN6 knockout flies. Whole body samples of 5 days old adult male flies were used for tRNA BS-seq. Sites covered by at least 10 reads in both samples and with a methylation level of ≥ 0.1 in either wild-type or mutant flies are shown.

(**D**) The sequence context flanking NSUN6-dependent m^5^C sites. Adult fly ovary samples were used for mRNA BS-seq and a total of 224 NSUN6-dependent sites (methylated in wild-type (≥ 0.1) but not mutant flies (< 0.05)) were identified.

(**E**) Metaprofiles of the secondary structure of NSUN6-dependent m^5^C sites and flanking regions in fly. Data were analyzed as in **Figure 1I**.

(**F**) NSUN6-tRNA structure in different species. Top: the overall structure of human, mouse, and fly NSUN6 in cartoon representation. Bottom: the binding of tRNA to NSUN6 in human, mouse, and fly. Human NSUN6 structure (PDB: 5wws) was from Liu et al [[2](#_ENREF_2)]. General RosettaCM protocol [[3](#_ENREF_3)] was used to predict mouse and fly NSUN6 structures.


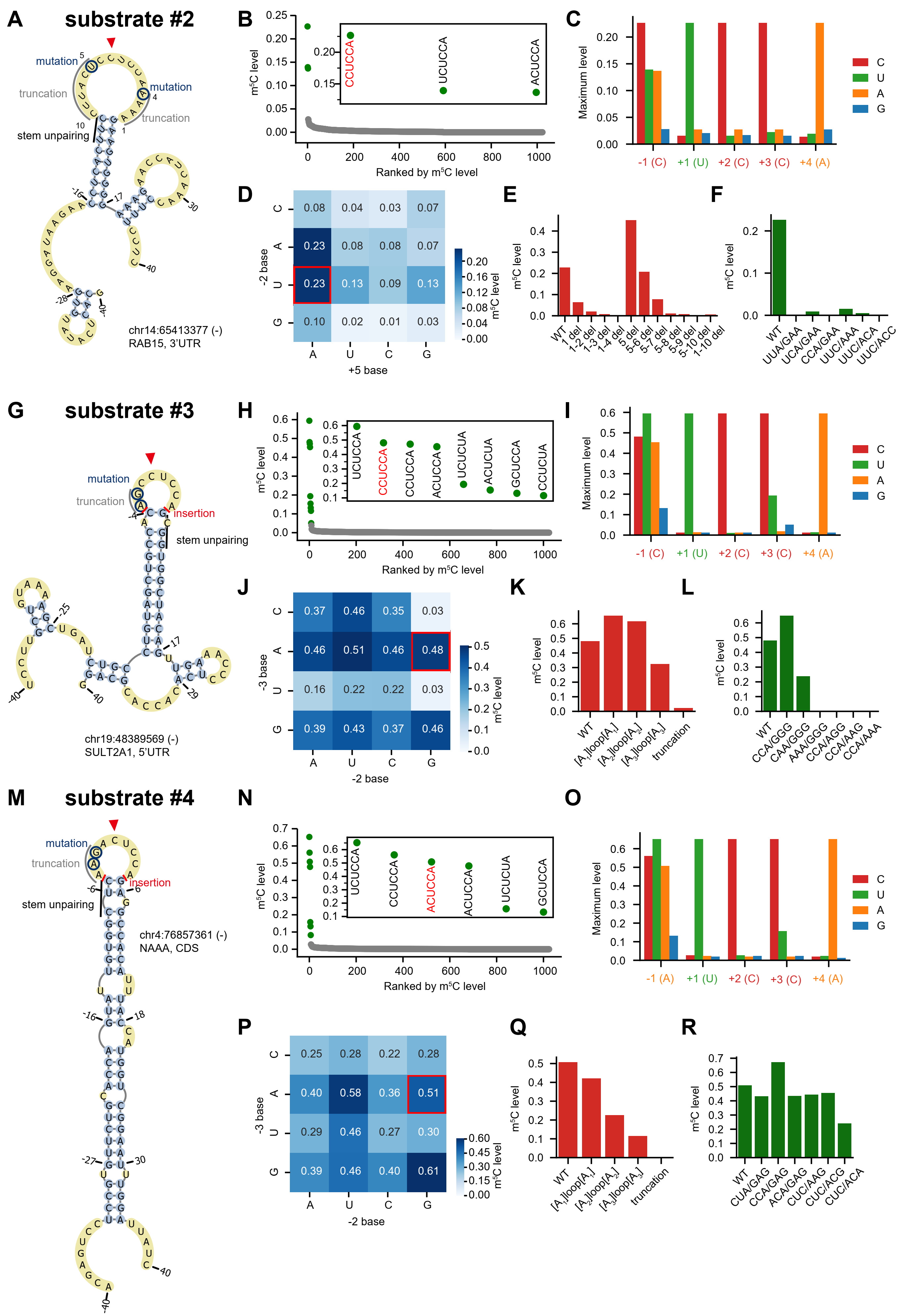


**Figure S5. Validation of the sequence and structural requirement for NSUN6-mediated mRNA methylation with additional three substrates.**

High-throughput mutagenesis assay analysis for additional three substrates. (**A-F**) substrate #2, chr14:65413377 (-) in RAB15 3’UTR; (**G-L**) substrate #3, chr19:48389569 (-) in SULT2A1 5’UTR; (**M-R**) substrate #4, chr4:76857361 (-) in NAAA CDS. (**A/G/M**) The predicted structure of the substrate. The m^5^C site is indicated by the red arrowhead. The modification types of the substrate are highlighted: loop mutation, blue; loop truncation, gray; base insertion, red; stem unpairing, black. (**B/H/N**) The methylation levels of variants with core motif (Nm^5^CUCCA) mutations. The original motif of the substrate is colored in red. (**C/I/O**) Barplot showing the maximum methylation levels of the substrates with mutations in the core motif. The original bases are indicated in parentheses. (**D/J/P**) Heatmap showing the methylation levels in different loop mutations. The original bases are highlight in red. (**E/K/Q**) The methylation levels of the substrates with truncation or extension of the loop region. For substrate #2, the positions of the deleted bases are indicated by numbers in **A**. (**F/L/R**) The methylation levels of the substrates of which the base-pairing of the stem regions were disrupted. To disrupt the stem, C-to-A, U-to-C, G-to-A, and A-to-C mutations were introduced.


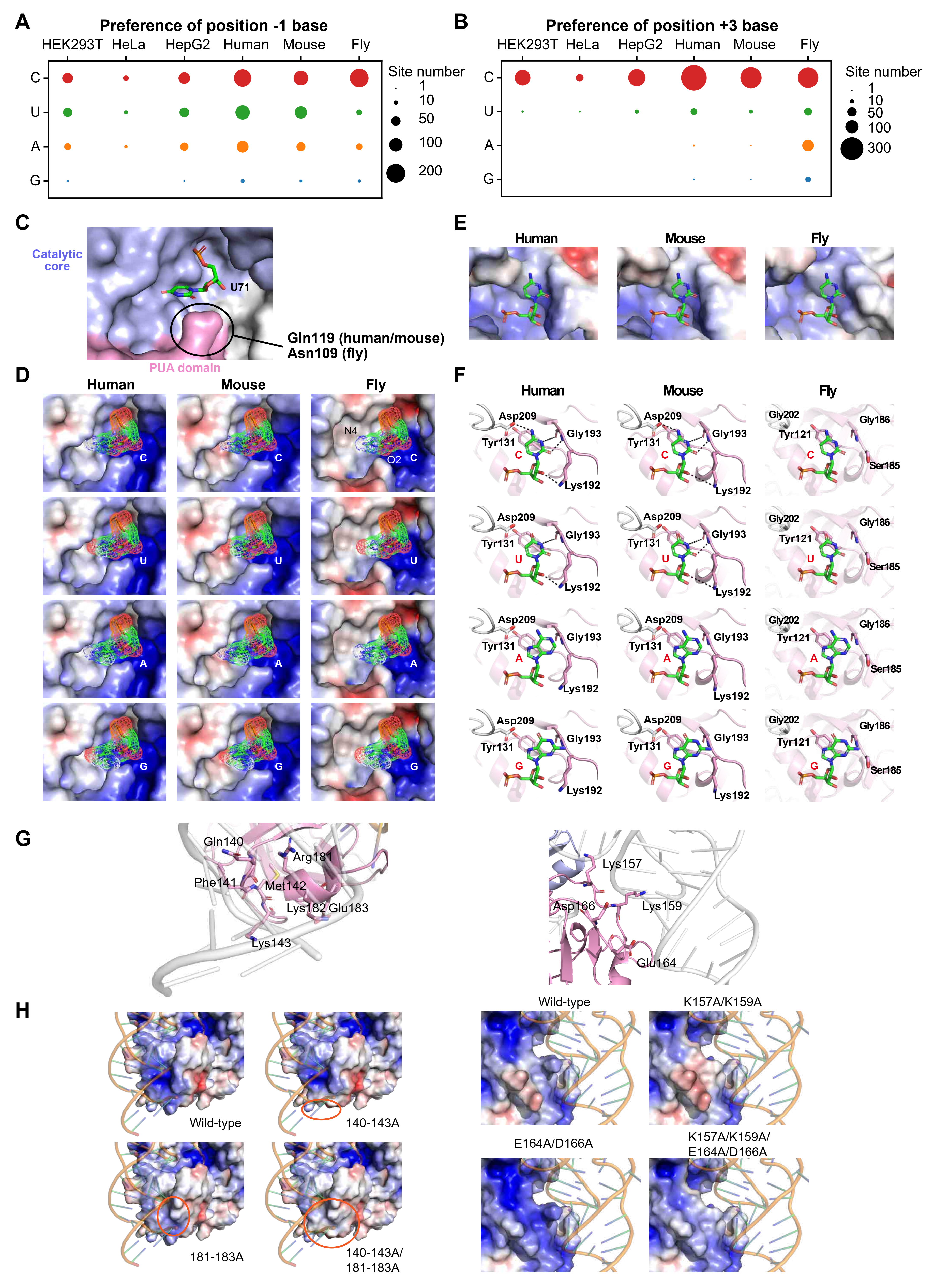


**Figure S6. Analysis of NSUN6-RNA interactions.**

(**A**) The number of m^5^C sites with different base compositions at position -1. m^5^C sites identified in HEK293T cells, HeLa cells, HepG2 cells, human pooled adult tissue data, mouse pooled adult tissue data, and fly pooled sample data were analyzed.

(**B**) The number of m^5^C sites with different base compositions at position +3.

(**C-D**) The position -1 base and its surrounding electrostatic surface. Human NSUN6 structure was adopted from Liu et al. Mouse and fly protein structures were generated by comparative modelling. In human and mouse, steric hindrance may occur when the pyrimidines were replaced by purines (especially G), whose carbonyl group might be repulsed by the pocket. In fly, bases at position -1 are located in a more negatively charged pocket, and C may be the most favorable base in this pocket because it has a positive charge amino group towards the pocket surface and a negative charge carbonyl group towards Asn109 in fly NSUN6 (homologous to Gln119 in human).

(**E**) The position +3 base and its surrounding electrostatic surface. Human NSUN6 structure was adopted from Liu et al. Mouse and fly protein structures were generated by comparative modelling.

(**F**) The interactions between position +3 bases and NSUN6 residues. Possible interactions are indicated using black lines.

(**G**) The residues selected to be mutated in **Figure 3A**.

(**H**) The possible electrostatic surface change after replacing selected PUA domain residues by Ala.


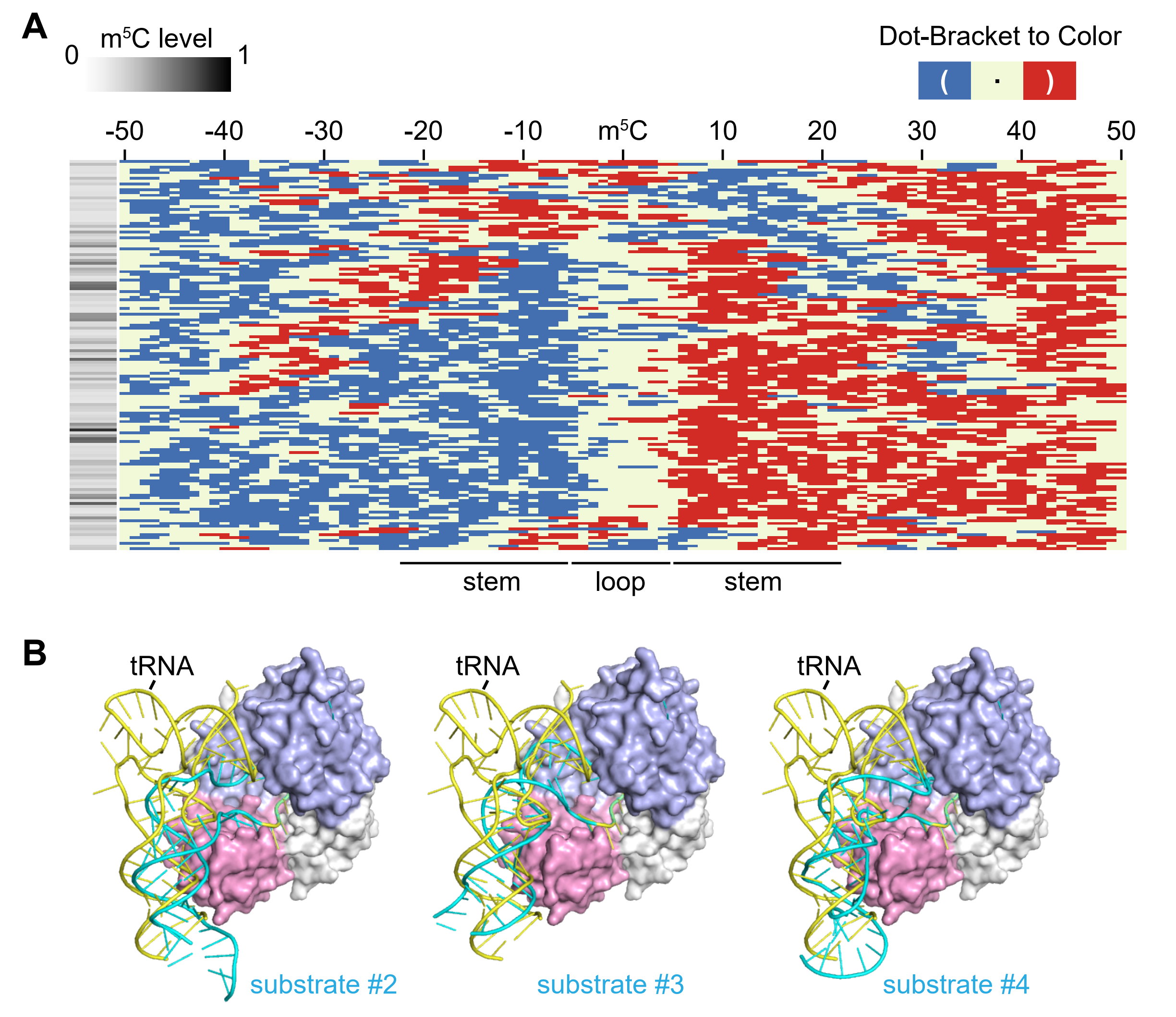


**Figure S7. NSUN6-mRNA structure prediction.**

(**A**) Meta-plot showing the secondary structures of individual Type II sites and flanking regions. The m^5^C levels of the sites in HEK293T cells are also indicated.

(**B**) A proposed NSUN6-mRNA structural model in a tRNA-like mode, along with NSUN6-tRNA co-crystallization structure (PDB: 5wws). tRNA is colored in yellow and mRNA is colored in blue. Three different NSUN6 mRNA substrates were used for the prediction.


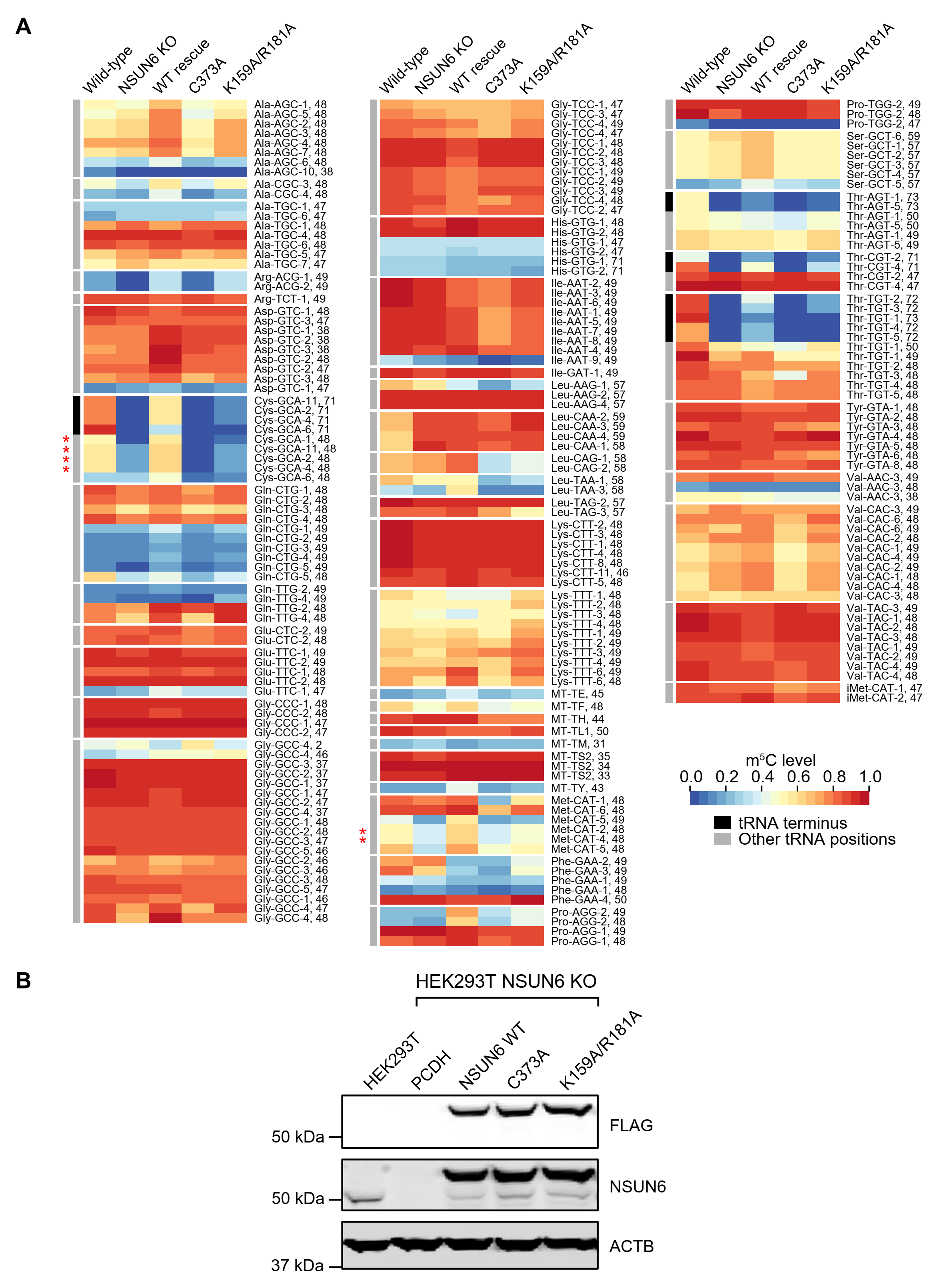


**Figure S8. tRNA methylation profiles in different samples.**

**(A)** Comparison of tRNA methylation profiles between different samples. Heatmap showing the tRNA methylation profiles in wild-type cells and NSUN6 knockout cells rescued by wild-type NSUN6 or variants. Non-terminus positions that lose methylation in the NSUN6 knockout cells are marked by the asterisk. Sites covered by at least 10 reads in all samples and with a methylation level of ≥ 0.1 in wild-type cells are shown.

(**B**) Western blot analysis of the NSUN6 proteins in wild-type cells or NSUN6 knockout cells overexpressed with different variants individually. ACTB served as a loading control.


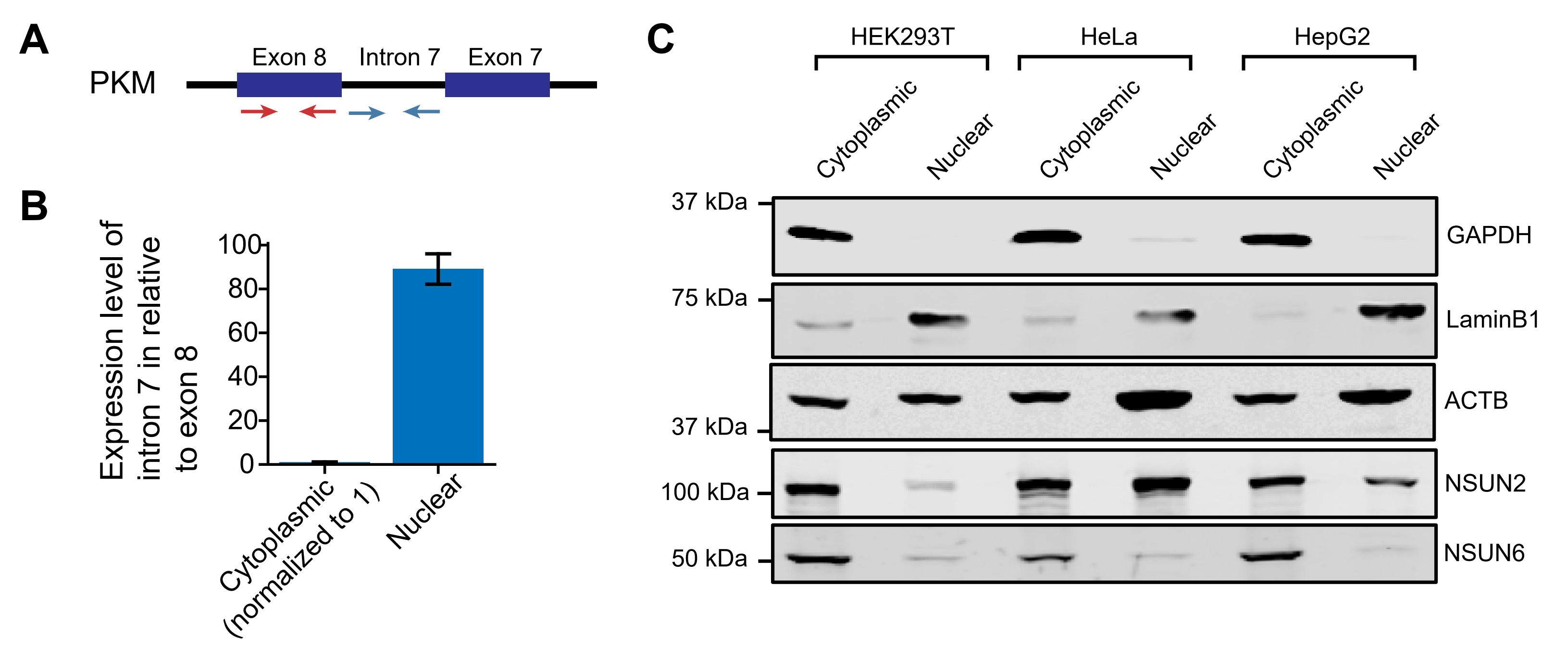


**Figure S9. Characterizing the deposition of Type II and Type I m^5^C sites.**

(**A-B**) qPCR validation of cytoplasmic and nuclear RNA separation. In brief, we randomly selected a gene expressed in HEK293T cells based on ENCODE data and confirmed that intron 7 reads were only observed in nuclear RNA-seq data set (data not shown). Next, we designed two primer pairs to amplify exon 8 (red arrows) and intron 7 (blue arrows) (**A**) and performed qPCR using cytoplasmic and nuclear RNA fractions. Finally, the expression level of intron 7 was first normalized to exon 8 in each fraction, and then the relative expression of intron 7 in nuclear fraction as compared to that in cytoplasmic fraction (set to 1) was calculated.

(**C**) Western blot verification of cytoplasmic and nuclear fraction separation in HEK293T cells, as well as the western blot analysis of NSUN2 and NSUN6 expression in cytoplasmic and nuclear fractions of HEK293T, HeLa and HepG2 cells. GAPDH, cytosolic marker; LaminB1, nuclear marker.


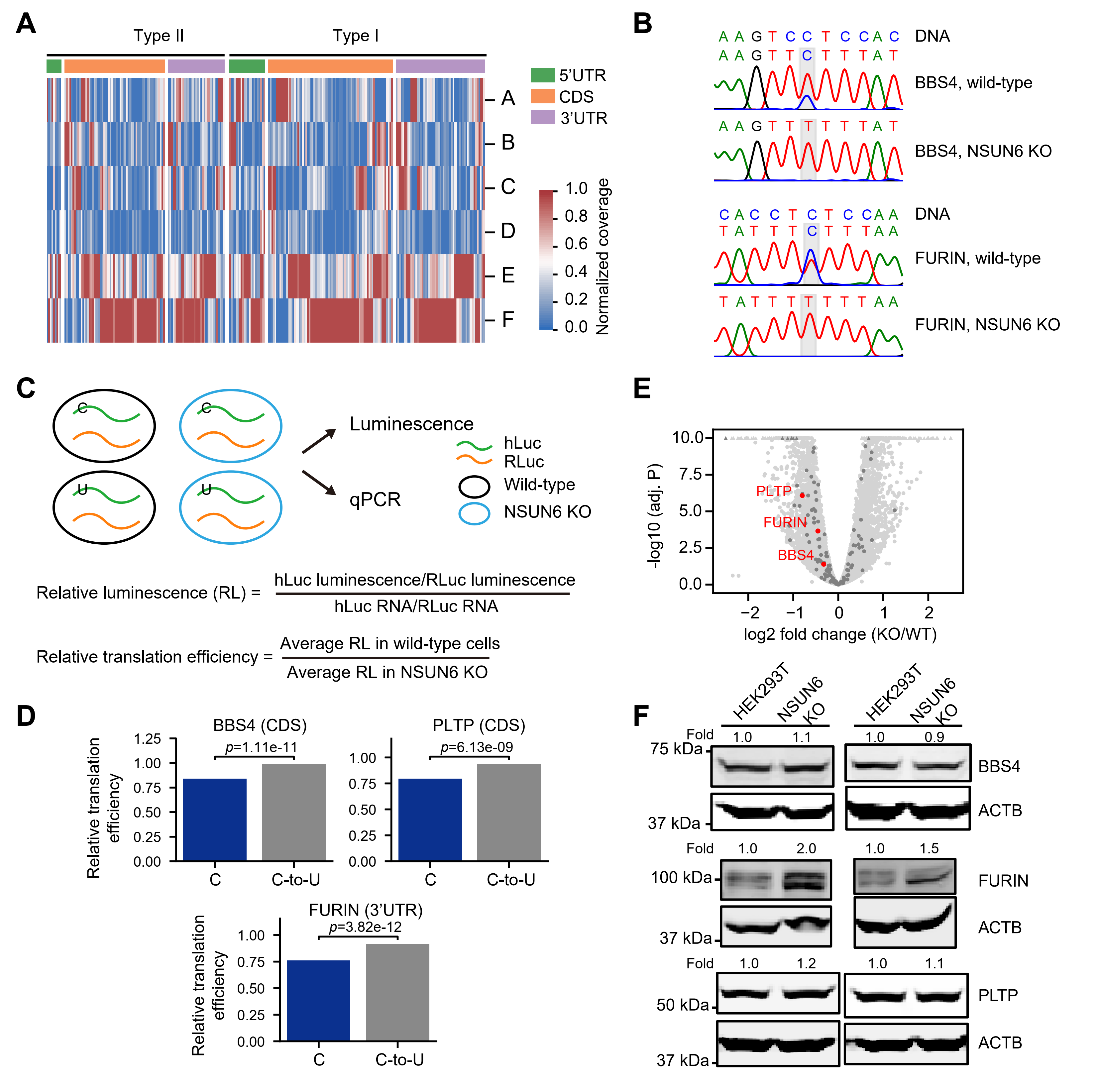


**Figure S10. A weak negative correlation between mRNA m^5^C and translational efficiency.**

(**A**) Normalized read coverage of m^5^C sites across the polysomal fractions. Vertical lines represent methylated Type I or Type II m^5^C sites. The data were Min-Max normalized as in **Figure 4F**. 106 Type II and 154 Type I m^5^C sites with at least 20 reads in at least 5 fractions are shown.

(**B**) BS-PCR followed by Sanger sequencing of PCR amplicons shows that the transiently expressed reporter genes were methylated. Notably, due to the presence of homopolymer (polyU, C-to-U converted RNA) in both upstream and downstream of PLTP m^5^C site, we were not able to evaluate the methylation status of this reporter gene.

(**C**) Illustration of the dual-luciferase reporter assay used to examine the impact of a single m^5^C site on translational control.

(**D**) Four Type II m^5^C sites (3 CDS sites and 1 3’UTR site) were examined (n = 3 independent experiments). The p values were calculated using the two-way ANOVA test (P-values of F-test of base factor (C and C-to-U) in two-way ANOVA test). The source data are provided in **Table S8**.

(**E**) Comparison of gene expression levels between NSUN6 knockout and control HEK293T cells via RNA-seq. Genes containing m^5^C sites selected for the reporter assay are highlighted in red.

(**F**) Western blot showing the protein levels of selected genes in NSUN6 knockout and control HEK293T cells. Two biological replicates were performed.

**METHODS**

**Cell culture**

HEK293T, HeLa and HepG2 cell lines were purchased from Cell Bank, Type Culture Collection, Chinese Academy of Sciences (CBTCCCAS). All cell lines have been identity verified using short tandem repeat (STR) analysis, which involves the simultaneous amplification of 17 STR markers plus amelogenin to confirm the identity of the cells, by CBTCCCAS. Cell lines were maintained in DMEM (Gibco) supplemented with 10% FBS (HyClone). All cell lines have been checked for mycoplasma contamination by CBTCCCAS and are routinely tested for mycoplasma by PCR detection of conditioned medium.

**Fly husbandry**

Fly stocks were kept at 25 °C on a 12 hours light and 12 hours dark cycle and fed cornmeal food.

**NSUN6 plasmid construction**

To construct NSUN6 expression plasmid, cDNA from HEK293T cells was reverse transcribed using HiScript II Q RT SuperMix (Vazyme), and the full-length NSUN6 CDS fragment was amplified. The NSUN6 fragment was inserted into the AvrII and AgeI sites of the pCDH-3xFLAG vector to generate pCDH-3xFLAG-NSUN6 plasmid. NSUN6 mutagenesis was performed using Mut Express II Fast Mutagenesis Kit V2 (Vazyme) or CloneExpress Ultra One Step Cloning Kit (Vazyme).

**Knockout cell generation and rescue experiment**

NSUN2 knockout, NSUN6 knockout, and NSUN2/NSUN6 double knockout cells were generated via CRISPR/Cas9-induced mutagenesis. In brief, sgRNA sequences were designed using CRISPR-ERA (http://CRISPR-ERA.stanford.edu) (**Table S5**). The sgRNA template oligonucleotides were synthesized and cloned into lentiCRISPR v2 plasmid (Addgene#52961). The plasmid was transfected into the cells using Lipofectamine 3000 (Thermo Fisher Scientific) following the manufacturer’s instruction. Transfected cells were selected using puromycin (1μg /ml). Mutant clones were selected by Sanger sequencing.

For the rescue experiment, knock out cells were plated in a 6-well plate. 3 μg of NSUN6 plasmid was transfected using Lipofectamine 3000 following the manufacturer’s instruction. 48 hours after the transfection, proteins of the cells were collected to confirm the expression of NSUN6 by western blot.

**Nuclear/cytoplasmic fraction**

HEK293T cells were fractionated using the PARIS kit (Ambion) according to the manufacturer’s instruction. The separation of cytoplasmic and nuclear fractions was validated using qPCR and western blot. For qPCR, the cytoplasmic and nuclear RNAs were reverse transcribed using HiScript II Q RT SuperMix (Vazyme), and the qPCR was performed using ChamQ SYBR qPCR Master Mix (Vazyme) with the following primers: the exon 8 of PKM: AGAGCATGATCAAGAAGCCCC (forward) and GAGGATAGTCCCCTTTGGCTG (reverse); the intron 7 of PKM: ACACTCGCATGTTTGTATGGG (forward) and TGTTACGTGCGACAATTCCA (reverse). For western blot, GAPDH and LaminB1 were used as cytosolic and nuclear markers, respectively.

***Drosophila* genetics and sample collection**

*Drosophila melanogaster* W1118 was used as wild-type control. The mutant allele for NSUN6 was generated using the CRISPR/Cas9-induced mutagenesis system following the previously described procedure [[4](#_ENREF_4)]. In brief, a sgRNA sequence was designed using flyCRISPR Optimal Target Finder (http://www.flyrnai.org/crispr/) (**Table S5**). The sgRNA template oligonucleotide was synthesized and cloned into the donor vector pUAST-attB. The sgRNA-pUAST-attB plasmid was microinjected into 86Fb (Bloomington 24749) embryos. The microinjection was performed by Core Facility of Drosophila Resource and Technology, SIBCB (CAS). The sgRNA transgenic line was crossed with vas-Cas9, so the progeny expressed both sgRNA and vas-induced Cas9 in the germline. The genotype of F1 was screened by Sanger sequencing.

**Construction of NSUN6 substrate plasmids**

For low-throughput experiments, the substrate template oligos were synthesized and individually cloned into the psiCHECK-2 vector using the seamless cloning method with CloneExpress Ultra One Step Cloning Kit (Vazyme). Plasmids were extracted with the endotoxin-free plasmid extraction kit (TIANGEN).

For high-throughput experiments, the ~140 bp substrate pools were synthesized at BGI Shenzhen. The fragments were amplified, gel purified, and inserted into psiCHECK2 vector by Gibson assembly. The reactions were performed using NEBuilder HiFi DNA Assembly Master Mix (NEB) by mixing the fragments with the linearized vector in a 7:1 molar ratio. 4 µl assembled products were used for bacteria transformation. Bacteria were shaken in SOC medium in 250 rpm for 60 minutes, and then separated on 14 cm LB agar ampicillin selective plates. After 37 ℃ incubation for about 12 hours, 8 plates of bacteria were harvested. Plasmids were extracted with the endotoxin-free plasmid extraction kit (TIANGEN).

**Analysis of cell viability and apoptosis**

For cell viability analysis, we used the CellTiter 96 AQueous One Solution Cell Proliferation Assay kit (Promega) according to the manufacturer’s instruction. In brief, 1 x 10^3^ wild-type or knock out cells were seeded into each 96-well plate. 0, 24, 48 and 72 hours after the seeding, 20 μl One Solution Reagent was added. The cells were incubated at 37 ℃ for 1 hour, and the absorbance at 490 nm was recorded.

For cell apoptosis analysis, Annexin V -FITC and Propidium iodide (PI) staining were used to determine the early apoptosis and cell death.

**RNA-seq**

For each sample, 1 μg total RNA was used for library construction. poly(A)+ RNA was separated from total RNA using Oligo dT Magnetic Beads (Vazyme). RNA was then used for library construction with NEBNext Ultra II Directional RNA Library Prep Kit (NEB). Libraries were sequenced on Illumina Hiseq X10 (Illumina) to produce paired-end 150 bp reads.

**mRNA BS-seq**

mRNA BS-seq library construction was performed as we previously described [[1](#_ENREF_1)]. In brief, total RNA was isolated with TRIzol reagent and Direct-zol RNA MiniPrep kit. poly(A)+ RNA was separated from total RNA using Oligo dT Magnetic Beads (Vazyme). 100 ng - 1 μg of poly(A)+ RNA was converted using the EZ RNA methylation kit (Zymo Research) with a modified high-stringency conversion condition (Sulfonation: 3 cycles, (1) 70 °C, 10 minutes. (2) 64 °C, 45 minutes. Desulfonation: 25 °C for 30 minutes.). The converted RNA was fragmented into 150 - 200 nt fragments by incubation at 94 °C for 8 minutes in fragmentation buffer (NEB). The fragmented RNA was then used for library construction using NEBNext Ultra II Directional RNA Library Prep Kit. SYBR Green I was added to the PCR mixture and the amplification was done with a qPCR instrument so that the amplicons can be monitored at each cycle to avoid unnecessary extra amplification. Libraries were sequenced on Hiseq X10 (Illumina) to produce paired-end 150 bp reads. All libraries are summarized in **Table S1**.

**BS-PCR and targeted BS-seq**

Total RNA was extracted with TRIzol reagent and Direct-zol RNA Kit (Zymo Research) 48 hours after the transfection. Total RNA was treated with DNase I, BS converted (Sulfonation: 1 cycle, (1) 70 °C, 10 minutes. (2) 64 °C, 45 minutes. Desulfonation: 25 °C for 30 minutes.), and reverse transcribed with gene-specific primers using HiScript II Q RT SuperMix (Vazyme). Target sequences were amplified using STARmix Taq DNA Polymerase (GenStar) with the following program: 94 °C for 3 minutes; 30 cycles of 94 °C for 30 seconds, 52 °C for 30 seconds and 72 °C for 20 seconds; and 72 °C for 1 minute. For BS-PCR, Sanger sequencing of PCR amplicons was applied to measure the methylation level of the mRNA substrate. For targeted BS-seq or targeted sequencing of plasmid pools, the first round of PCR was performed with 25 cycles using the same program above. Next, the PCR product was purified with DNA Clean Beads (Vazyme) and dissolved in 10 µl water. 1 µl purified product was amplified using sequencing adaptors with the same program above for 5 cycles. Finally, the PCR product was recovered with Zymoclean Gel DNA Recovery Kit (Zymo Research) and sequenced. All primers used for targeted BS-seq are listed in **Table S6**.

**tRNA BS-seq**

20 µg total RNA was first loaded into 15% denaturing TBE-Urea PAGE Gels and separated based on the molecular weight. The gel slice corresponding to 70-90 nucleotides was excised from the PAGE gel and the RNA fragments were recovered using ZR small-RNA PAGE Recovery Kit (Zymo Research) following the manufacturer’s protocol. RNA was then converted using the EZ RNA methylation kit (Zymo Research) as we previously described [[1](#_ENREF_1)]. End-repair was performed with T4 PNK (NEB) to phosphorylate 5’-hydroxyl termini and remove 2’,3’-Cyclic phosphate produced during the bisulfite/desulfonation reaction. The tRNA BS-seq library was generated using VAHTS Small RNA Library Prep Kit for Illumina (Vazyme) according to the manufacturer’s protocol. Libraries were sequenced on Hiseq X10 (Illumina) to produce paired-end 150 bp reads. All libraries are summarized in **Table S1**.

**Polysome RNA BS-seq**

Cells were collected and lysed with lysis buffer (10 mM Tris-HCl (pH 7.4), 5 mM MgCl_2_, 100 mM KCl, 1% Triton X-100, 2 mM DTT, 100 μg/ml CHX (CST), 50 U/ml RiboLock RNase Inhibitor (Thermo Fisher Scientific) and cOmplete, EDTA-free Protease Inhibitor Tablets (1 tablet per 10ml, Roche)). 10–50% sucrose gradients were prepared in gradient buffer (20 mM HEPES-KOH, pH7.4, 5 mM MgCl_2_, 100 mM KCl, 2 mM DTT, 100 μg/ml CHX, 50 U/ml RiboLock RNase Inhibitor) using a Gradient Master (Biocomp). 400 μl lysates were loaded on the gradients. Gradients were centrifuged at 36,000 rpm for 2 hours at 4 ℃ in an SW-40 Ti rotor and then fractionated using the Gradient Master. RNA was equally divided into 22 fractions and these fractions were mixed: fraction 1-4, free RNA, with 0 ribosome; fraction 5-6, RNA with ribosome small subunit and 0 ribosome; fraction 7-8, RNA with ribosome large subunit and 0 ribosome; fraction 9-11, monosome; fraction 12-17, light polysome, with 2-5 ribosomes; fraction 18-22, heavy polysome, with >5 ribosomes. RNA was then isolated using TRIzol reagent. poly(A)+ RNA of each fraction was separated from total RNA using Oligo dT Magnetic Beads (Vazyme). About 10-50 ng mRNA of each fraction was converted using the EZ RNA methylation kit (Zymo Research) with a modified high-stringency conversion condition. The converted RNA was then used for library construction with NEBNext Ultra II Directional RNA Library Prep Kit. Libraries were sequenced on Hiseq X10 (Illumina) to produce paired-end 150 bp reads. All libraries are summarized in **Table S1**.

**Ribo-seq**

Active ribosome protected fragments (RPFs) were isolated using the RiboLace Ribo-Seq kit (Immagina Biotechnology) according to the manufacturer’s instruction. Next, rRNA was depleted with biotinylated oligos. rRNA-depleted RPFs were then treated with T4 PNK (NEB), and the library was generated using VAHTS Small RNA Library Prep Kit for Illumina (Vazyme). For the RNA-seq control, poly(A)+ RNA was separated from total RNA and the library was constructed with NEBNext Ultra II Directional RNA Library Prep Kit (NEB).

**Dual-luciferase reporter assay**

The wild-type fragments were amplified with Phusion High-Fidelity DNA Polymerase (NEB) using HEK293T cDNA. For each of the sites in CDS, a fragment in the m^5^C-containing gene in-frame (BBS4,90 bp; PLTP, 81 bp) was inserted right behind the start codon of the Firefly luciferase [[5](#_ENREF_5)]. For the site in 3’UTR, a 160 bp fragment harboring the m^5^C site was inserted into downstream of the Renilla coding region. The C-to-T point mutation was introduced using Mut Express II Fast Mutagenesis Kit V2 (Vazyme). Plasmids were transfected into NSUN6 knockout or wild-type HEK293T cells using Lipofectamine 3000. Renilla and Firefly luminescence was measured 24 hours later using Dual-Glo Luciferase Assay System (Promega) on GloMax -96 Microplate Luminometer (Promega). All primers used to construct the reporter genes and quantify the expression of Renilla and Firefly luciferases are listed in **Table S7**.

**Genome assembly and gene models**

Genome, transcriptome and gene annotations of human GRCh37.75, mouse GRCm38.85 and fly BDGP5.78 were downloaded from Ensembl. tRNA isodecoder sequences were downloaded from GtRNAdb [[6](#_ENREF_6)]. “CCA” tails were added before index build. rRNA sequences were downloaded from SILVA database (release 138) [[7](#_ENREF_7)].

**mRNA BS-seq data analysis**

mRNA m^5^C sites were called as we previously described [[1](#_ENREF_1)]. We first trimmed adapters, the first 10 bp of the reads, the last 6 bp of the reads, and the low-quality bases using Cutadapt (-e 0.25 -q 25 -trim-n) [[8](#_ENREF_8)] and Trimmomatic [[9](#_ENREF_9)]. Then clean reads were mapped to the *in silico* converted genome by HISAT2 (-k 10,–fr,–rna-strandness FR,–no-mixed) [[10](#_ENREF_10)] to obtain unique alignments. The remaining unmapped and multiple mapped reads were further mapped to the *in silico* converted transcriptome by Bowtie2 (-end-to-end,–fr,–gbar 5,–mp 5, -k 10, -R 2, -D 5) [[11](#_ENREF_11)]. Alignment results were merged together, and only bases with high quality (Q ≥ 30) were used for the variant calling. Last, the sites were called using a series of filters as previously described [[1](#_ENREF_1)]. In brief, we inspected all positions with C-to-T mismatches and only took variant positions into consideration if they conformed to our requirements for number, frequency, and quality of bases that vary from the converted reference sequences: (i) each variant is supported by 3 or more variant nucleotides having a base quality score of ≥ 30, mismatch frequency ≥ 0.1 and coverage of C+T ≥ 20; (ii) the variant still satisfies the above criteria after the removal of the overlapped C-reads based on the Gini coefficient determined C-cutoff filter; (iii) the signal ratio of the variant is ≥ 0.9; (iv) the variant is not located at conversion-resistant genes and (v) the p-value calculated using one-sided binomial test based on gene-specific conversion rate is < 0.001. The methylation level (mismatch frequency) is defined as the number of reads with C divided by the number of reads with C or T.

**Targeted BS-seq data analysis**

Reads were mapped to the reference sequences (DNA library against the original reference sequences; BS-seq library against the C-to-T converted reference sequences) with Bowtie2 (--norc). Custom scripts were used to extract reads with barcodes that are unique in both DNA and BS-seq libraries. The C/T counts at m^5^C position were extracted and the methylation level is defined as the number of reads with C divided by the number of reads with C or T.

**tRNA BS-seq data analysis**

Adapters were first trimmed with Cutadapt (--max-n 1 -m 18 -e 0.25 --trim-n -q 20) [[8](#_ENREF_8)]. Only reads containing adapters were retained. Clean reads were first C-to-T (read 1) or G-to-A (read2) converted and mapped to C-to-T converted reference sequences containing tRNA, mRNA, and rRNA with Bowtie2 (--end-to-end --no-mixed --norc -k 50 -X 80). Read pairs were ranked by alignment scores (sum of AS and YS tags in the BAM file). Only read pairs with the highest alignment score and uniquely mapped to one tRNA type (multiple alignment on tRNA isodecoders was allowed) were used. Last, original reads were recovered with a custom script and piled up to call m^5^C sites and calculate their methylation levels. This method ensures us to discover the loss of methylation at isodecoder level, although it is imprecise in detecting the increase of methylation due to the possible cross-alignment.

**Motif analysis**

The m^5^C sites and flanking regions were extracted from the transcriptome (exonic sites) or the genome (intronic sites). Motif logos were plotted with WebLogo [[12](#_ENREF_12)].

**m^5^C density calculation**

To generate the metagene profile of m^5^C site distribution across transcripts, 5’UTR was fixed to 5 bins and then CDS and 3’UTR were divided into bins based on their relative average lengths to 5’UTR (human CDS, 25 bins; human 3’UTR, 20 bins; mouse CDS, 30 bins; mouse 3’UTR, 30 bins; fly CDS, 30 bins; fly 3’UTR, 15 bins). Last, m^5^C density in each bin was defined as the ratio of m^5^C number to the total C number.

**Background C generation**

Background cytosines mean Cs adjacent to a UCCA motif on m^5^C-containing genes. Background sites were randomly selected by the bootstrap method. In brief, Cs with UCCA motif were randomly selected from transcripts with Type II m^5^C sites (e.g. if a transcript had 2 m^5^C, 2 Cs were sampled). Then the sequence flanking those sampled Cs were folded. This step was repeated 100 times to calculate the median and quartile for each of the bases.

**Analysis of miCLIP data**

NSUN6 (GSE66011) and NSUN2 (GSE44386) miCLIP data were obtained from GEO [[13](#_ENREF_13), [14](#_ENREF_14)]. 3’ adapters were trimmed with Cutadapt (-m 18 -q 20 --trim-n). Possible 5’ adapters were also trimmed. Clean reads were first mapped to rRNAs (Silva-release132 and Ensembl rRNA and mt-rRNA) with Bowtie2 (--norc -N 0 -L 20). Unmapped reads were further mapped to tRNAs (GtRNAdb and Ensembl tRNA and mt-tRNA). The remaining unmapped reads were mapped to the reference genome and transcriptome (Ensembl release 75) with HISAT2 and only uniquely mapped reads were used for binding site identification in mRNAs. For NSUN2 data, 9%, 81% and 10% of the reads were mapped to rRNA, tRNA, and mRNA, respectively; for NSUN6 data, 31%, 40% and 29% of the reads were mapped to rRNA, tRNA, and mRNA, respectively.

For mRNA binding site identification, the miCLIP truncation sites (5’ ends of aligned reads) within protein-coding gene exons (2,2810 genes for Ensembl release 75) were assigned to the nearest cytosine at ±2nt regions (priority: 0, -1, +1, -2, +2), as previously described [[13](#_ENREF_13)]. The priority of assignment is: 0, -1, +1, -2, +2. FDR was calculated as previously described [[15](#_ENREF_15)] but without crosslinking site extension. To generate a background, truncation sites within a gene were randomized and then assigned to the nearest cytosine. Cytosines with FDR < 0.05 were considered as NSUN6 binding sites. For NSUN6 data, sites shared in all replicates were used for analysis. For NSUN2 data, due to the limited number of mapped reads in mRNAs, sites identified in each replicate were merged for analysis. Notably, since these are overexpression experiments, caution is needed when interpreting the results.

**Comparative modelling of mouse and fly NSUN6**

General RosettaCM protocol [[3](#_ENREF_3)] was used in NSUN6 structure prediction. The protein part of NSUN6-tRNA complex (PDB: 5wws) was used as the template. Mouse (ENSMUSG00000026707) and fly NSUN6 (FBgn0037200) protein sequences were first aligned to human NSUN6 (ENSG00000241058) by MUSCLE [[16](#_ENREF_16)]. 3- and 9-mer fragments of mouse or fly NSUN6, as well as the predicted secondary structure, were generated by Robetta (http://robetta.bakerlab.org/fragmentsubmit.jsp). Then threading protocol was run with minirosetta. 1,000 structures generated by random seeds were ranked and the best ones were selected.

**Protein-mRNA complex structure prediction**

NSUN6-mRNA complex structure prediction was performed as previously described [[17](#_ENREF_17)]. Crystal structure of human NSUN6- tRNA^Cys^(GCA) (G2A:C71U) complex (PDB: 5wws) was used as the initiating model. Based on the knowledge learning from NSUN6-tRNA co-crystallization structure and the computational analysis we performed, we required that both NSUN6 and Um^5^CUCCA (position -1 to +5) were fixed and the upstream and downstream sequences of Um^5^CUCCA is double stranded. RNA substrate sequence (ucugagggaggaggccaccucuccaagggcuucugcacccuccaccc) and secondary structure [...(((((.(((((((.(........))))))))...)))))......] were passed to rnp_denovo program of Rosetta (version 3.10), with the parameters of -new_fold_tree_initializer true -minimize_rna true -rna:denovo:lores_scorefxn rna/denovo/rna_lores_with_rnp_aug.wts -rna_protein_docking true -ignore_zero_occupancy false -convert_protein_CEN false -FA_low_res_rnp_scoring true -cycle 1000. Over 20,000 structures were generated. RNA parts of these structures were additionally scored by rna_score in Rosetta. Structures with both high complex score and high RNA score were passed for further refinement (several bases were extended in this step) and we selected the substrates with RNA substrate stem interacts with the PUA domain.

**RNA structure prediction and illustration**

RNA secondary structure was predicted using RNAfold (2.4.12) [[18](#_ENREF_18)] with default settings. The RNA structure in **Figure 2A** was drawn by PseudoViewer 3 web application [[19](#_ENREF_19)].

**Protein structure illustration**

Human NSUN6-tRNA co-crystallization data by Liu et al.[[2](#_ENREF_2)] were downloaded from Protein Data Bank (PDB: 5wws). Protein annotation of NSUN6 was referred to Liu et al. Protein alignment and structure visualization were performed with PyMOL (http://www.pymol.org/). The electrostatic surface was predicted with APBS electrostatics [[20](#_ENREF_20)].

**RNA-seq analysis**

Adapters were first trimmed with Cutadapt [[8](#_ENREF_8)]. The clean reads were mapped to the reference genome by Tophat2 [[21](#_ENREF_21)] in strand-specific manner (--library-type fr-firststrand). Alignments were then processed to HTSeq-count [[22](#_ENREF_22)] to obtain read counts of each gene. Finally, differential gene expression analysis was performed with DESeq2 [[23](#_ENREF_23)].

**TE calculation**

TE was calculated as previously described [[24](#_ENREF_24)], with some modifications. We first preprocessed the FASTQ files before mapping by trimming the adapters, filtering low-quality bases, discarding reads < 20nt and shortening reads to 26nt (cutadapt -m 20 -l 26 -q 25). Clean reads were then mapped to rRNA sequences by Bowtie2[[11](#_ENREF_11)] (-N 1 -L 20 --norc) to eliminate rRNA contamination. rRNA sequences were downloaded from SILVA database (https://www.arb-silva.de/). The remaining reads were then mapped to the reference genome and transcriptome by Tophat v2.1.1 [[21](#_ENREF_21)] (-M -N 1). To avoid ribosome stalling around start codon, the first 16 codons were discarded and only ORF with ≥ 40 codons were used [[25](#_ENREF_25)]. Only uniquely mapped reads were selected and processed to HTseq-count for feature counting.

To quantify mRNA or RPF abundance, genes with ≥ 60 mapped reads were selected and normalized using Trimmed Mean of M values (TMM) method implemented in the edgeR Bioconductor package [[26](#_ENREF_26)]. TE was calculated by dividing TMM normalized RPF value to that of gene expression value. Only genes with ≥ 60 mapped reads in the mRNA-seq data were used.

**Supplementary Notes**

**Note S1. The UCCA motif is a robust signature to** **distinguish NSUN2-dependent sites from NSUN2-independent sites.**

To utilize the UCCA motif alone to distinguish NSUN2-dependent sites from NSUN2-independent sites, it is required that NSUN2-dependent sites do not have the UCCA motif, and meanwhile, NSUN2-independent sites contain the UCCA motif. To ask whether this is the case, we analyzed mRNA BS-seq of NSUN2 knockout HeLa and HEK293T cells. We found that NSUN2-dependent and -independent sites had the G-rich triplet and UCCA motifs, respectively, in both HeLa and HEK293T cells (**Figure S1A**). Moreover, nearly no NSUN2-dependent sites had a UCCA motif and most NSUN2-independent sites contained a UCCA motif (**Figure S1B**). Thus, the UCCA motif is a robust signature for Type II m^5^C site identification with low false-assignment rate of Type I m^5^C sites.

**Note S2. Estimating the proportion of NSUN6-dependent m^5^C sites in HEK293T cells based on knockout data.**

To calculate the proportion of NSUN6-dependent m^5^C sites in HEK293T cells, we compared the methylation profiles between wild-type and NSUN6 knockout cells. NSUN6-dependent sites were defined as sites that 1) were methylated in wild-type cells (mismatch frequency ≥ 0.1, coverage of C+T ≥ 20 and p < 0.001), 2) had coverage of C+T ≥ 20 in knockout cells, and 3) had mismatch frequency < 0.05 in knockout cells. Of the 281 m^5^C sites identified in wild-type cells and with enough depth in knockout cells, 140 sites had a mismatch frequency < 0.05 in knockout cells. Thus we estimated that 49.8% of m^5^C sites were NSUN6-dependent.

**Note S3. The impact of NSUN6 on tRNA methylation.**

Position C72 of different isodecoders of tRNA^Thr-TGT^, tRNA^Thr-CGT^, tRNA^Thr-AGT^ , and tRNA^Cys-GCA^ had different responses in the K159A/R181A rescue experiment (**Figure S8A**). Isodecoders such as tRNA-Cys-GCA-10 and tRNA-Cys-GCA-6, which have an identical or similar D-stem as the tRNA^Cys-GCA^ substrate used in the previous experiment [[2](#_ENREF_2)], completely lost their methylation in K159A/R181A mutant rescue cells. However, very low-level methylation was detected in other isodecoders. This may be because other isodecoders have a subtle difference in the complex conformation, and the previous experiment only reflects the details of a subset of the isodecoders. Hence, further NSUN6 design is needed to eliminate the terminus methylation in all isodecoders. Interestingly, both increased and decreased methylation levels of non-terminus Cs were also found in the NSUN6 knockout cells (**Figure 3C** and **Figure S8A**). For example, we found that, in position C48 of tRNA^Cys-GCA^, the methylation was lost in the NSUN6 knockout cells (**Figure 3C** and **Figure S8A**). This result suggests that NSUN6 may indirectly affect the methylation of non-NSUN6-target sites, possibly by competitive inhibition of other methyltransferases or affecting the tRNA processing pathway. Further studies are required to investigate the mechanisms.

**REFERENCES**

1. Huang T, Chen W, Liu J*, et al*: Genome-wide identification of mRNA 5-methylcytosine in mammals. *Nat Struct Mol Biol* 2019, **26**:380-388.

2. Liu RJ, Long T, Li J*, et al*: Structural basis for substrate binding and catalytic mechanism of a human RNA:m5C methyltransferase NSun6. *Nucleic Acids Res* 2017, **45**:6684-6697.

3. Song Y, DiMaio F, Wang RY*, et al*: High-resolution comparative modeling with RosettaCM. *Structure* 2013, **21**:1735-1742.

4. Ren X, Yang Z, Xu J*, et al*: Enhanced specificity and efficiency of the CRISPR/Cas9 system with optimized sgRNA parameters in Drosophila. *Cell Rep* 2014, **9**:1151-1162.

5. Safra M, Sas-Chen A, Nir R*, et al*: The m1A landscape on cytosolic and mitochondrial mRNA at single-base resolution. *Nature* 2017, **551**:251-255.

6. Chan PP, Lowe TM: GtRNAdb 2.0: an expanded database of transfer RNA genes identified in complete and draft genomes. *Nucleic Acids Res* 2016, **44**:D184-189.

7. Quast C, Pruesse E, Yilmaz P*, et al*: The SILVA ribosomal RNA gene database project: improved data processing and web-based tools. *Nucleic Acids Res* 2013, **41**:D590-596.

8. Martin M: Cutadapt removes adapter sequences from high-throughput sequencing reads. *EMBnetjournal* 2011, **17**.

9. Bolger AM, Lohse M, Usadel B: Trimmomatic: a flexible trimmer for Illumina sequence data. *Bioinformatics* 2014, **30**:2114-2120.

10. Kim D, Langmead B, Salzberg SL: HISAT: a fast spliced aligner with low memory requirements. *Nat Methods* 2015, **12**:357-360.

11. Langmead B, Salzberg SL: Fast gapped-read alignment with Bowtie 2. *Nat Methods* 2012, **9**:357-359.

12. Crooks GE, Hon G, Chandonia J-M*, et al*: WebLogo: A Sequence Logo Generator. *Genome Res* 2004, **14**:1188-1190.

13. Van Haute L, Dietmann S, Kremer L*, et al*: Deficient methylation and formylation of mt-tRNA(Met) wobble cytosine in a patient carrying mutations in NSUN3. *Nat Commun* 2016, **7**:12039.

14. Hussain S, Sajini Abdulrahim A, Blanco S*, et al*: NSun2-Mediated Cytosine-5 Methylation of Vault Noncoding RNA Determines Its Processing into Regulatory Small RNAs. *Cell Reports* 2013, **4**:255-261.

15. König J, Zarnack K, Rot G*, et al*: iCLIP reveals the function of hnRNP particles in splicing at individual nucleotide resolution. *Nature Structural & Molecular Biology* 2010, **17**:909-915.

16. Edgar RC: MUSCLE: multiple sequence alignment with high accuracy and high throughput. *Nucleic Acids Res* 2004, **32**:1792-1797.

17. Kappel K, Das R: Sampling Native-like Structures of RNA-Protein Complexes through Rosetta Folding and Docking. *Structure* 2019, **27**:140-151.e145.

18. Lorenz R, Bernhart SH, Höner zu Siederdissen C*, et al*: ViennaRNA Package 2.0. *Algorithms for Molecular Biology* 2011, **6**:26.

19. Byun Y, Han K: PseudoViewer3: generating planar drawings of large-scale RNA structures with pseudoknots. *Bioinformatics* 2009, **25**:1435-1437.

20. Jurrus E, Engel D, Star K*, et al*: Improvements to the APBS biomolecular solvation software suite. *Protein Sci* 2018, **27**:112-128.

21. Kim D, Pertea G, Trapnell C*, et al*: TopHat2: accurate alignment of transcriptomes in the presence of insertions, deletions and gene fusions. *Genome Biol* 2013, **14**:R36.

22. Anders S, Pyl PT, Huber W: HTSeq-a Python framework to work with high-throughput sequencing data. *Bioinformatics* 2014, **31**:166-169.

23. Love MI, Huber W, Anders S: Moderated estimation of fold change and dispersion for RNA-seq data with DESeq2. *Genome Biol* 2014, **15**:550.

24. Park JE, Yi H, Kim Y*, et al*: Regulation of Poly(A) Tail and Translation during the Somatic Cell Cycle. *Mol Cell* 2016, **62**:462-471.

25. Artieri CG, Fraser HB: Accounting for biases in riboprofiling data indicates a major role for proline in stalling translation. *Genome Res* 2014.

26. Robinson MD, McCarthy DJ, Smyth GK: edgeR: a Bioconductor package for differential expression analysis of digital gene expression data. *Bioinformatics* 2010, **26**:139-140.
